# Reverse Phase High Performance Liquid Chromatography Method for the Estimation of Mangiferin in Nano Formulations: Development and Optimization by Analytical Quality-by-Design

**DOI:** 10.1021/acsomega.5c12935

**Published:** 2026-03-14

**Authors:** Akshatha P. Kamath, Lalit Kumar, Srinivas Mutalik, Pawan Ganesh Nayak, Krishnadas Nandakumar

**Affiliations:** † Department of Pharmacology, Manipal College of Pharmaceutical Sciences, 76808 Manipal Academy of Higher Education (MAHE), Manipal, Karnataka 576104, India; ‡ Department of Pharmaceutics, National Institute of Pharmaceutical Education & Research, Hajipur, Bihar 844 102, India; § Department of Pharmaceutics, Manipal College of Pharmaceutical Sciences, 76808 Manipal Academy of Higher Education (MAHE), Manipal, Karnataka 576104, India

## Abstract

A novel reverse-phase high-performance liquid chromatography (RP-HPLC) method was developed and optimized for the estimation of Mangiferin (MGF) by employing a Quality by Design (QbD) approach. Preliminary method screening was done by the Taguchi OA, followed by using a Box–Behnken Design (BBD), enabling systematic evaluation of critical factors with a reduced number of experimental runs. The model efficiently established significant correlations between selected variables and analytical responses, thereby enhancing the method’s robustness and reliability. Chromatographic separation was achieved on a Hyperclone C18 column (4.6 × 250 mm, 5 μm) using an isocratic mobile phase consisting of phosphate buffer (pH 3.2) and acetonitrile (30%) + methanol (70%) in a 78:22% v/v ratio, at a flow rate of 1.0 mL/min. Detection was performed using a photodiode array detector at 258 nm. The method was validated in accordance with ICH Q2­(R2) guidelines. Validation parameters, including system suitability, linearity, accuracy, precision, robustness, sensitivity, and solution stability, were found within acceptable limits. The proposed method was successfully applied for the analysis of the stability study of MGF and to analyze the release kinetics of MGF SLNs, formulated via high-pressure homogenization and the sonication technique. The results confirm the applicability of the developed RP-HPLC method for routine quality control of combination nanocarrier systems.

## Graphical abstract

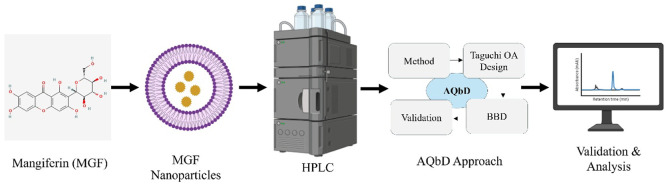

## 1 Introduction

Neurodegenerative diseases like Alzheimer’s Disease (AD) are characterized by the gradual deterioration of neurons, resulting in cognitive decline and memory loss. Alzheimer’s, the most prevalent neurodegenerative disorder, currently affects more than 55 million people globally, with numbers expected to rise significantly as the global population ages. Current treatments only address symptoms and do not stop or reverse disease progression, mainly due to challenges such as limited blood-brain barrier penetration and the complex nature of the disease.^1,2^ In recent years, phytochemicals have emerged as promising alternatives due to their ability to target multiple pathways, provide neuroprotection, and offer a favorable safety profile.^3,4^

Mangiferin (MGF) is a naturally occurring C-glycosylxanthone polyphenol, with high concentration in the mango tree (*Mangifera indica*) and other plants.^5,6^ MGF displays a broad spectrum of pharmacological properties, such as antioxidant, anti-inflammatory, antidiabetic, anticancer, and neuroprotective activities.^7–15^ Though it has significant therapeutic potential, the clinical use of MGF is limited by its low solubility, permeability, and bioavailability. The oral bioavailability of MGF is only 1.2%. Many literatures affirm that the preparation of lipid-based formulations increases the solubility and bioavailability of the drugs. Hence, preparing a lipid-based formulation, such as solid lipid nanoparticles, is a novel approach to increase the bioavailability of the drug.^16,17^ Limited literature is available on HPLC techniques used for MGF estimation, which are associated with significant limitations, such as low sensitivity with linearity limited to the microgram range, gradient flow, incomplete validation, high solvent consumption, and long retention time (Table 1).^18^

**Table 1:** Different Other Methods to Detect Mangiferin Reported Previously

Sl. No	Mobile phase composition	Stationary phase and detection wavelength	LOD and LOQ	Retention time	Drawbacks	Refs
1.	Phosphoric acid solution (0.5%): acetonitrile (gradient elution)	•Didmonsil C18 column	•LOD: -	•-	•Gradient elution can be more complex and time-consuming.	^23^
		•Detection at 320 nm	•LOQ: -		•Microgram linearity range	
2.	Acetonitrile: orthophosphoric acid (0.2% OPA) (10:90)	•Prontosil C18 column	•LOD: -	•14.6 min	•Acidic conditions could affect column stability	^24^
		•UV detection at 242 nm	•LOQ: -		•Delayed Rt	
					•LOD and LOQ not determined	
3.	Methanol: 2.5% aqueous solution of acetic acid v/v (28:72%, v/v) and acetic acid aqueous solution (3%): methanol (70:30%, v/v)	•LiChrospher RP 18	•LOD: 1.98 ng	•5.5 min	•Acidic conditions could affect column stability	^25^
		•UV detection at 254 nm	•LOQ: 6.2 ng	•7.6 min	•Narrow robustness evaluation	
					•Limited application testing	
					•Restricted stability evaluation	
4.	0.1% Formic acid: acetonitrile (87:13)	•Kinetex XB-C18	•LOD: 0.48 ppm	•6.129 min	•Higher flow rate may reduce column life	^18^
		•UV at 256 nm	•LOQ: 1.95 ppm		•Poor sensitivity	
		•Flow: 1.5 mL/min			•Limited validation data	
5.	Water: methanol: glacial acetic acid (55:26:0.5 v/v)	•C18 column	•LOD: -	•3.46 min	•Rt is too less that comes within 5 min	^26^
		•254 nm	•LOQ: -		•LOD and LOQ not determined	
					•Narrow sensitivity range	
					•Incomplete validation	
					•No forced degradation studies performed	
6.	Acetonitrile: buffer (Phosphoric acid 0.03% v/v) 45:55 v/v	•PhenomenexC18 column	•LOD: 2.53 μg/mL	•4.76 min	•Low flow rate (0.5 mL/min)	^27^
		•254 nm	•LOQ: 7.66 μg/mL		•Microgram linearity range	
					•No forced degradation studies performed	

The Analytical Quality by Design (AQbD) framework has gained considerable attention for the development of robust and reliable analytical methods by enabling a systematic understanding of Critical Method Parameters (CMPs) that influence method performance.^19^ As a science and risk-based strategy, AQbD facilitates the identification and assessment of CMPs that affect critical analytical attributes (CAAs) through tools such as risk assessment and factor screening studies. This is followed by an evaluation of potential interactions among these variables using Design of Experiments (DoE) to achieve optimized and reproducible method outcomes. The AQbD methodology encompasses defining a Quality Target Method Profile (QTMP) and relevant CAAs, selecting key CMPs based on their potential impact, and applying appropriate experimental designs, including response surface methodology (RSM), to model and locate the optimal method conditions.^20,21^ This process leads to establishing a well-defined analytical design space and developing a suitable control strategy to ensure consistent method performance and continuous improvement.

Numerous studies have demonstrated the effectiveness of AQbD in developing cost-effective, efficient liquid chromatographic methods for the quantification of diverse analytes in both pharmaceutical formulations and biological matrices.^22^ In the current study context, the AQbD approach was specifically applied to the HPLC method development for MGF, aiming to identify and control method variables exhibiting high variability and significant influence on performance. As a result, the present investigation was directed toward establishing a simple, robust, sensitive, and economical AQbD-based HPLC method for the accurate quantification of MGF in analytical samples, as existing methods may not provide sufficient sensitivity for detecting low concentrations of MGF, especially in complex samples. Additionally, the choice of mobile and stationary phases significantly impacts the performance of HPLC methods, and developing new HPLC designs can enhance detection limits and improve the overall sensitivity of the method. The purpose of this study was to develop a sensitive and cost-effective analytical method, validate it according to ICH guidelines, and apply it to the *in vitro* dissolution analysis of formulated Mangiferin solid lipid nanoparticles (MGF-SLNs).

## 2 Materials and Methods

### 2.1 Chemicals and Reagents

Mangiferin (MGF, 95% purity) (Cat. no: 4773-96-0) was purchased from Nanjing NutriHerb BioTech Co., Ltd. (China). HPLC-grade acetonitrile (Cat. no: 70030LC250), methanol (Cat. no: 30930LC250), and potassium dihydrogen orthophosphate (KH_2_PO_4_) (Cat. no: 7778-77-0) were obtained from Merck Specialties Pvt. Ltd. (Mumbai, India). Triethylamine (TEA) (Cat. no: 121-44-8) was bought from Spectrochem Ltd. (India). Milli-Q water was obtained from Sartorius Arium Mini Ultrapure Water System (Sartorius AG, Göttingen, Germany). All other reagents and solvents used were of HPLC grade.

### 2.2 Equipment

A liquid chromatography system (SIN-20ACHT, Shimadzu Corporation, Kyoto, Japan) equipped with a binary pump, an autosampler, and a photodiode array (PDA) detector set at 258 nm was employed for method development and validation. Chromatographic data were collected and processed using LC Solution Software (v.5.57). The experimental design studies were performed with Design-Expert software (v.13.0; Stat-Ease Inc., Minneapolis, MN, USA).

### 2.3 Analytical Conditions

Extensive trials with buffer, pH and mobile phase composition, and stationary phase the MGF separation was carried with an isocratic mode using Chromatographic column Phenomenex (HyperClone 5 μm ODS (C18) 120 Å, LC Column 250 × 4.6 mm) as stationary phase and mobile phase as mixture of methanol and acetonitrile (70:30) (Phase A) and 10 mM KH_2_PO_4_ (Phase B) with 0.1% TEA and pH was adjusted to 3.2 ± 0.05 using Orthophosphoric acid (OPA, 1% v/v).

### 2.4 Standard Solution and Working Stock Preparation

The primary stock solution (1,000 μg/mL) was prepared by dissolving 1 mg of the drug in 1 mL of 80% methanol, followed by bath sonication for 10 min. The secondary stock solution of 100 μg/mL was diluted from the primary stock. Different concentrations of working stock solutions, 50, 100, 150, 300, 500, 1000, 3000, 4000, and 5000 ng/mL, were prepared in methanol using the secondary stock solution aliquots.

### 2.5 Method Development by AQbD Concept

#### 2.5.1 Analytical Target Profile (ATP)

The first step in method development was to define the Analytical Target Profile (ATP). The objective was to develop an HPLC method for de-encapsulating MGF from lipid nanocarriers with minimal interference from excipients, matrix components, and degradation products, and to perform the analysis within an acceptable run time. The ATP specifically targeted optimization of chromatographic parameters to enhance method performance, achieving high specificity, minimal peak tailing, and reliable quantification of MGF in lipid nanocarrier formulations.^28^ The ATP further emphasized method robustness and reproducibility to ensure reliable quantification of MGF in lipid nanocarrier formulations throughout the analytical concentration range in in vitro dissolution studies as well.

#### 2.5.2 Critical Analytical Attributes (CAAs)

Critical Analytical Attributes (CAAs) are method-related factors that have a significant influence on method quality and are directly linked to the ATP. In AQbD-based method development, the key steps include defining the QTMP and identifying the CMPs that affect the CAAs to achieve improved method performance. Typical CMPs considered during AQbD optimization include flow rate, column oven temperature, mobile phase composition, and mobile phase pH. Common CAAs, in contrast, involve parameters such as peak area, Retention time (Rt), Number of Theoretical Plates (NTP), and Tailing factor (Tf).

#### 2.5.3 Risk Assessment

Before optimizing chromatographic conditions, a risk assessment must be conducted. Risk assessment determines the probability of risks and failures associated with the most significant parameters of those CAAs and CMPs. The Ishikawa fishbone diagram was used to assess the impact of CMPs on CAAs, and a literature review and the supported preliminary experimental findings were included. The assessed parameters included mobile phase composition, buffer pH, buffer strength, flow rate, injection volume, detection wavelength, and column temperature. Based on the qualitative risk evaluation, parameters with a higher potential impact on CAAs were selected for further statistical investigation using design of experiments (DoE).

### 2.6 Design of Experiments (DoE)

Following the risk assessment, statistical experimental designs were employed to evaluate and optimize the selected parameters. The factors were screened using an L8 (2^7^) Taguchi Orthogonal Array (OA) design. Seven of the CPMs were included as mobile phase flow rate (X1), injection volume (X2), detection wavelength (X3), oven temperature (X4), mobile phase ratio (X5), buffer strength (X6), and Phosphate buffer pH (X7). The primary tool for model selection was using Pareto charts to determine statistically which of the several factors were affecting CAAs and compiled as dependent variables to determine which independent variable mainly contributed to the trends. The various levels of the experimental variables are detailed in Table S1, and the software-generated experimental runs are summarized in Table S2. Subsequently, a Box–Behnken Design (BBD) was implemented for further optimization of the most significant variables. This essentially involves three factors, each having two levels, and in return, has offered 17 experimental runs alongside five replicates of the center point. The replication of the center points helps to ascertain the reproducibility of the method and enhances the reliability of the experimental design. The critical independent variables selected for the HPLC method include the percentage of aqueous solvent, the pH, and the salt concentration in the aqueous phase (Table S3). At the same time, the NTP, peak area, and the Tf served as response variables. Data analysis was performed using Design Expert software.

### 2.7 Analytical Method Validation

The HPLC method was validated in accordance with ICH Q2­(R2) guidelines, assessing critical parameters such as specificity, linearity, accuracy, precision, detection and quantitation limits, robustness, and system suitability to ensure the method’s reliability and suitability for its intended analytical application.^29^

#### 2.7.1 System Suitability

To evaluate system suitability, consecutive injections (n = 6) of MGF at a concentration of 5000 ng/mL were injected into the HPLC system. For each injection, the peak area and Tf were measured. The mean values and the percentage relative standard deviation (%RSD) for both parameters were subsequently calculated to assess the system’s performance consistency.

#### 2.7.2 Specificity

To ensure specificity, we injected one blank sample, one blank SLN (formulation excipients without drug), and one MGF sample with the lowest concentration (50 ng/mL). We then recorded the Rt and peak area of the analyte and calculated the percentage of interference occurring at the analyte’s Rt in the blank sample.

##### 2.7.2.1 Stress Studies

Forced degradation studies were conducted according to ICH Q2­(R2) guidelines, including acid and base hydrolysis, oxidation, photodegradation, and thermal degradation. Increased stressor agent concentrations can accelerate the degradation rate, enable a rapid evaporation of the compounds, and raise MGF stability problems. Furthermore, forced degradation conditions are designed to hasten the degradation process to collect the data and analyze possible degradation pathways and products.^30^

##### 2.7.2.2 Hydrolysis

Acid hydrolysis was done using 0.1 and 1 N HCl, base hydrolysis was done using 0.1 N and 1 N NaOH, and neutral hydrolysis was assessed using Milli-Q water. 1 mg of MGF was weighed and dissolved in methanol to make a 1 mg/mL stock. From the primary stock, a secondary stock solution of 100 μg/mL was prepared, and 500 μL of secondary stock solution was mixed with 500 μL of each stressor and kept in different temperature conditions, like room temperature (RT), 4 °C, and 60 °C. 100 μL was withdrawn from each stressor at 0, 4, 8, 10, and 24 h, neutralized, and diluted accordingly to obtain a final concentration of 5 μg/mL prior to HPLC analysis.

##### 2.7.2.3 Oxidation

Oxidation was done using 3% H_2_O_2_ solution, mixed with the standard stock and kept in different conditions (RT, 4 °C, and 60 °C). 100 μL of the sample was withdrawn at specific time points (same as Acid hydrolysis) and diluted accordingly.

##### 2.7.2.4 Photodegradation

10 mg of MGF was weighed and kept under natural sunlight for photodegradation, and 1 mg of the sample was collected at each time point and diluted using a suitable diluent for analysis.

##### 2.7.2.5 Thermal Degradation

1 mg/mL of MGF was prepared in methanol and kept at 60 °C for thermal degradation. 100 μL of the sample was collected at each time point and diluted using a suitable diluent for the analysis.

#### 2.7.3 Linearity

Linearity was determined across a 50–5000 ng/mL concentration range, each analyzed in triplicate. The peak area for each concentration was plotted against the corresponding concentration to generate a calibration curve. Linear regression analysis provided the slope, intercept, and coefficient of determination (R^2^), confirming the method’s linear response across the tested range.

#### 2.7.4 Precision

The precision of the developed RP-HPLC method was assessed through intraday and interday studies at three concentration levels (150, 2500, and 4000 ng/mL) representing low, mid (100%), and high levels within the established linearity range of the method. All the samples at each concentration level were prepared from the same working solution to specifically evaluate analytical repeatability under controlled preparation conditions of the active compound. For intraday precision, six replicates of each level were analyzed in the morning and evening of a single day. Six replicates were analyzed on the mornings of two consecutive days for interday precision. The %RSD of peak areas was calculated for each concentration to evaluate repeatability and intermediate precision.

#### 2.7.5 Accuracy and Recovery

Accuracy of the developed RP-HPLC method was evaluated using the recovery approach at three concentration levels (75%, 100%, and 125%). Using the allegation method, test samples were prepared by spiking a known amount of the MGF standard (150 and 4000 ng/mL) to obtain final concentrations of 1875, 2500, and 3125 ng/mL. The reference standard solutions used for spiking were prepared from a 1000 μg/mL primary stock solution of MGF by appropriate dilution. The spiked samples were analyzed, and the measured concentrations of MGF were determined using the calibration curve. Percentage recovery was calculated based on the ratio of the measured concentration to the theoretical concentration added (eq 1). Accuracy was considered acceptable when the recovery was within 90–110% with %RSD ≤ 2%.

1 $$\mathrm{Percentage}\;\mathrm{recovery}=\frac{\mathrm{Measured}\;\mathrm{concentration}}{\mathrm{Theoritical}\;\mathrm{concentration}}\times 100$$

#### 2.7.6 Robustness

Robustness was tested by intentionally varying key chromatographic parameters: flow rate (1 ± 0.1 mL/min), aqueous-to-organic ratio (78:22 ± 2 units), buffer pH (3.2 ± 0.5), injection volume (10 ± 2 μL), and PDA wavelength (258 ± 2 nm). A 3000 ng/mL MGF sample was analyzed in triplicate for each condition. The %RSD for peak area and Tf was calculated to evaluate the method’s stability under slight procedural variations.

#### 2.7.7 Sensitivity (LOD and LOQ)

Sensitivity was evaluated by calculating the limits of detection (LOD) and quantitation (LOQ) based on the standard deviation of the response (σ) and the slope (s) of the calibration curve, using the following formulas (eq[Disp-formula eq2] & 3):

2 LOD=3.3σ/s

3 LOQ=10σ/s

### 2.8 Application of Validated HPLC Method

#### 2.8.1 Formulation of MGF-Loaded Solid Lipid Nanoparticles (MGF-SLNs)

Mangiferin-loaded solid lipid nanoparticles were prepared using a high-speed homogenization followed by probe sonication technique. Briefly, a predetermined amount of MGF was dispersed in the aqueous phase containing a stabilizing agent (2% w/v) under continuous stirring. In parallel, the lipid phase was prepared by dissolving the required quantity of lipids in an organic solvent. The lipid phase, prepared in an organic solvent, was added dropwise under high-speed homogenization, followed by probe sonication for 10 min under controlled conditions to reduce particle size. The resulting formulation was obtained as a stable colloidal suspension of lipid nanoparticles, which was further used for characterization.

#### 2.8.2 Estimation of Drug Content

From the formulated MGF nanoparticles, drug content was assessed by an indirect method, where 100 μL of the sample was taken and diluted 10 times using Milli-Q water. The diluted nanoparticle sample was centrifuged at 15000 rpm at 4 °C for 30 min to calculate the drug content. The supernatant was collected and loaded directly into the HPLC for analysis using the optimized analytical method. The study was done in triplicate, and the mean drug content was calculated using the equation.^31^

4 $$\%Entrapment\;Efficiency=\frac{\mathrm{Total}\;\mathrm{amount}\;of\;MGF-Free\;MGF\;\mathrm{amount}}{\mathrm{Total}\;MGF}\times 100$$

#### 2.8.3 In Vitro Drug Release

A dialysis bag diffusion method (MWCO of 12 kDa, HiMedia) was utilized for the in vitro drug release study. An accurately weighed amount of MGF-loaded nanoparticles equivalent to 1 mg of MGF, calculated based on drug loading, was filled into the dialysis bag separately, end of the dialysis bag was tied and sealed to prevent the leak the sample and placed in 100 mL of simulated gastric fluid (SGF) maintained at 37 ± 0.5 °C and agitated at 150 rpm. After 2 h, the dialysis bags were transferred to simulated intestinal fluid (SIF) for 6 h, followed by transfer to phosphate-buffered saline (PBS, pH 7.4) for the remaining duration of the study to simulate gastrointestinal transit conditions. Tween 80 (1% w/v) was added to each release medium to maintain sink conditions. 1 mL of samples collected at each time point (0, 0.5, 1, 2, 3, 4, 6, 8, 10, 12, 24, 48, and 72 h) and replaced with fresh media. Collected samples were loaded into the HPLC for analysis. The percentage of drug released was calculated using the regression equation from the MGF calibration curve. Several release kinetic models were used to predict the release process. The highest correlation coefficient value was used to determine which model was most appropriate for the release mechanism.

### 2.9 Statistical Analysis

Statistical analysis was done using the Design of Experiments software using One-Way ANOVA. All the values are represented as mean ± Standard Deviation (SD).

## 3 Results and Discussion

### 3.1 Method Development through Quality by Design

#### 3.1.1 Preliminary Method Development

Preliminary trials were conducted to develop an HPLC method for quantifying MGF based on the methods reported in the literature. From the literature, various organic solvents like acetonitrile, methanol, and aqueous solvents such as sodium acetate, formic acid, acetic acid, and phosphate buffers were employed at varying flow rates and mobile phase ratios. As MGF has multiple wavelengths, the detection wavelength 258 nm was fixed by using the UV–visible spectroscopic method. Accordingly, the mobile phase composition with ACN and methanol, with phosphate buffer and OPA as pH modifier, was used for further HPLC analysis.

#### 3.1.2 Risk Assessment

The interconnections between different input process variables, i.e, critical method parameters (CMPs) and their resulting critical analytical attributes (CAAs) were mapped using an Ishikawa (fishbone) diagram (Figure 1). Key input variables were identified and prioritized according to a risk management strategy, considering the level of risk each variable posed. The diagram illustrates the relationship between CMPs and CAAs. The selected CAAs included retention time, peak area, peak symmetry (tailing factor), and column efficiency, as these attributes directly influence analytical performance. Based on risk evaluation, flow rate, injection volume, mobile phase composition, buffer strength, pH, and detection wavelength were identified as high-risk CMPs. Medium or low risk parameters were maintained at constant values throughout the analysis.

**Figure 1:**
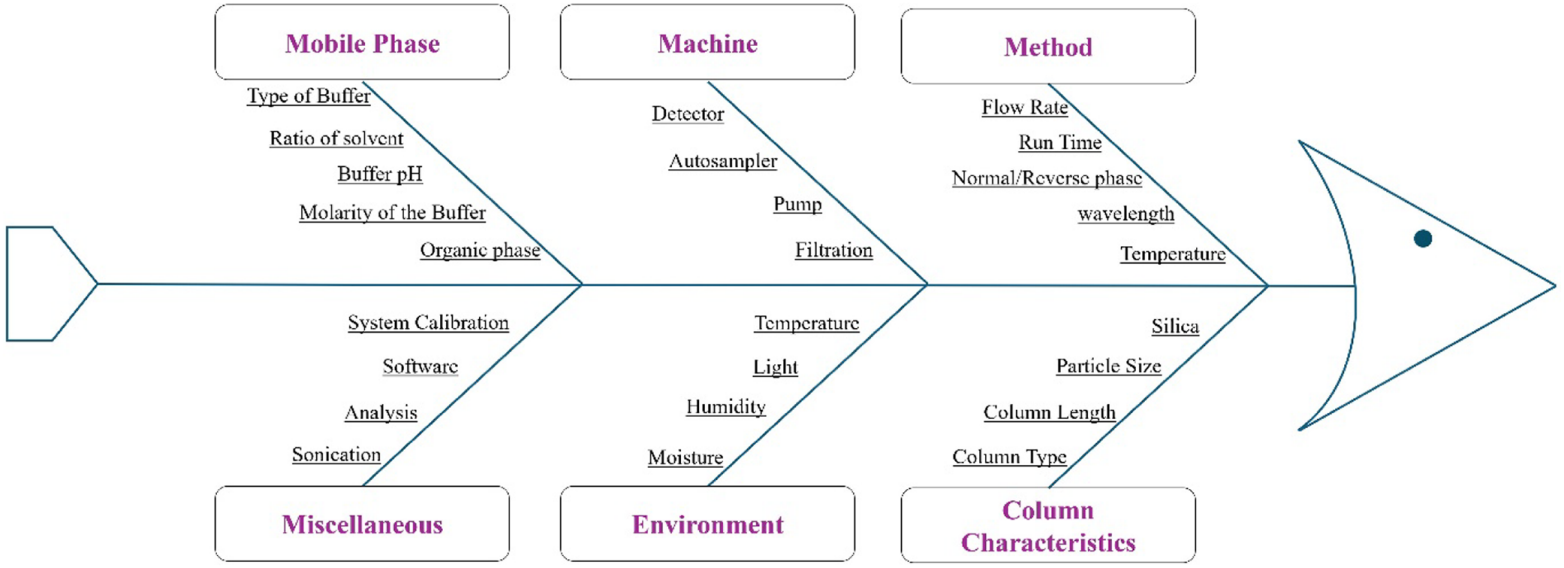
Fishbone (Ishikawa) diagram representing the risk factors involved in method development.

#### 3.1.3 Screening of Factors: Taguchi OA

The Taguchi OA design was chosen to evaluate the primary effects of CMPs on key CAAs in the HPLC method. The software recommended eight experimental runs to assess each CMP’s influence systematically. Responses from each run were recorded (Table 2) and analyzed for their main effects using one-way ANOVA and Pareto charts. According to the ANOVA results, the volume ratio Phosphate buffer (A), flow rate (B), and buffer pH (C) were found to have statistically significant effects (p < 0.05) and were therefore chosen for further optimization. The Pareto charts (Figure 2) further confirmed these variables as critical, as their bars exceeded the significance threshold, highlighting their importance in optimizing the HPLC method for MGF estimation.

**Table 2:** Experimental Results from Taguchi OA Design

Run	Flow rate (mL/min)	Inj. vol (μL)	Wavelength (nm)	Oven temp (°C)	Buffer ratio (%)	Buffer strength (mM)	Buffer pH	Retention time (min)	Peak area (mV.min)	NTP	Tailing factor
1	1.2	10	260	22	80	10	4	9.63	590373	2179.16	1.23
2	0.9	10	256	22	74	10	3	6.34	636715.66	2931.11	1.38
3	0.9	10	256	25	80	15	4	12.62	541271	3863.88	1.62
4	1.2	15	256	25	74	10	4	5.01	446168.33	2283.41	1.49
5	1.2	10	260	25	74	15	3	5.02	451942.33	3549.97	1.34
6	1.2	15	256	22	80	15	3	9.62	36753.333	2534.16	1.92
7	0.9	15	260	25	80	10	3	12.34	882576.66	2639.41	0.87
8	0.9	15	260	22	74	15	4	6.63	448174.66	1546.15	1.52

**Figure 2:**
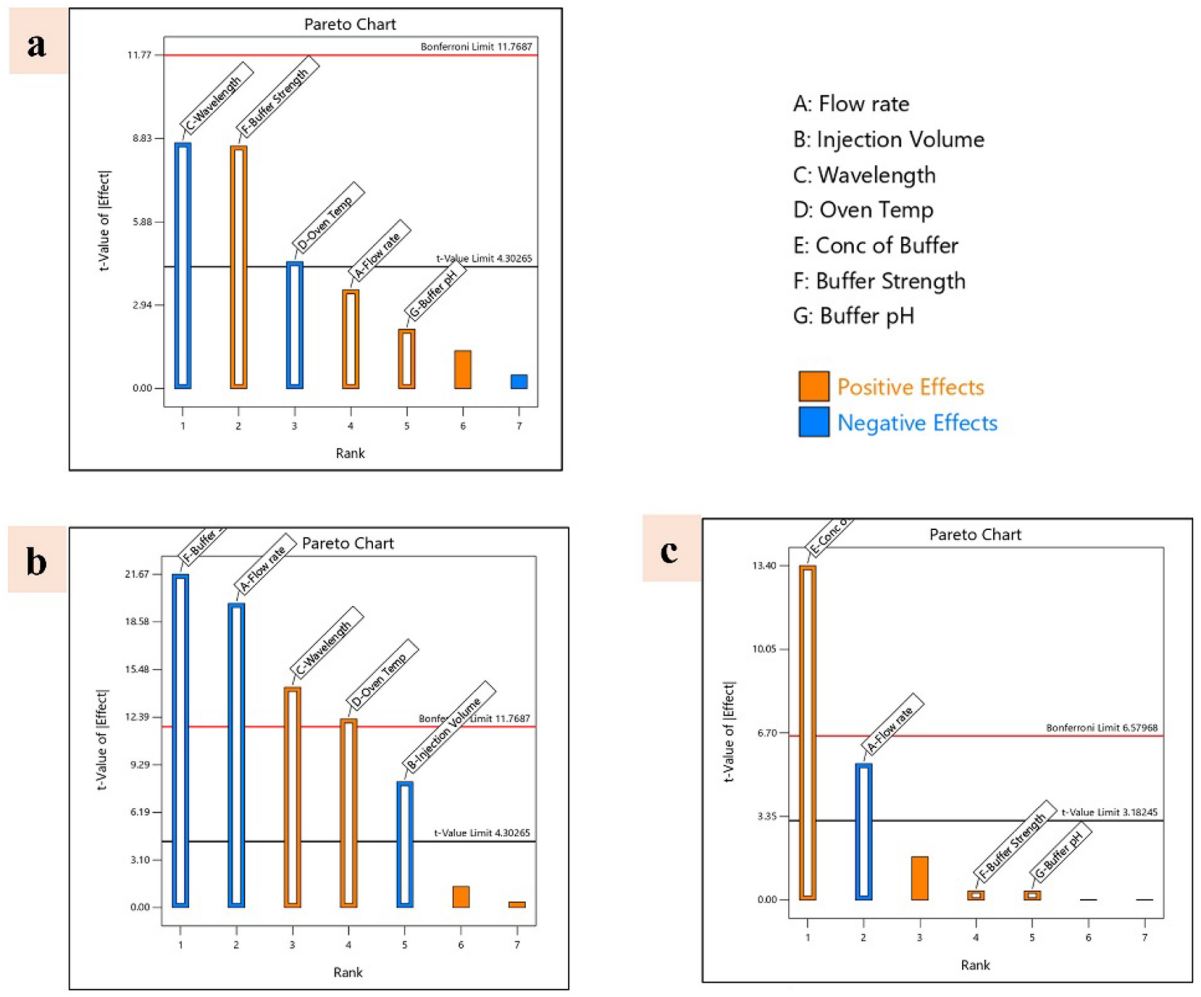
Pareto charts representing the responses on the factors (a) tailing factor, (b) peak area, (c) retention time.

### 3.2 Optimization by Box–Behnken Design (BBD)

Based on the results of the screening design, the volume ratio of phosphate buffer (A), flow rate (B), and buffer pH (C) were identified as CMPs that directly influenced the selected CAAs (Responses 1, 2, 3, and 4). Therefore, these CMPs were optimized to achieve the desired CAAs in line with the ATP of the HPLC method. Optimization was conducted using a DoE approach, specifically response surface methodology with a Box–Behnken Design (BBD). Design-Expert software generated 17 experimental runs that were executed in the laboratory, with the collected data subsequently entered into the software (Table 3). The results were analyzed through response surface methodology using ANOVA, multiple regression, and contour plots for interpretation.

**Table 3:** Table Representing BBD Experimental Runs with Corresponding Responses to Each Run

Run	F1 - Flow rate (mL/min)	F2 - Buffer ratio (%)	F3 - Buffer pH	R1 - Retention time (min)	R2 - Peak area (mV.min)	R3 - NTP	R4 - Tailing factor
1	1.2	74	3.5	5.11	421903	2543.44	1.35
2	1.2	74	3.5	5.09	421549.33	2538.72	1.36
3	1.2	78	3.8	7.56	391773.00	3217.98	1.41
4	1.4	74	3.2	4.25	378454.33	2464.35	1.36
5	1.0	74	3.2	5.91	514184.66	2710.84	1.35
6	1.4	70	3.5	3.35	367844.66	2052.17	1.41
7	1.4	74	3.8	4.36	356111.66	2275.87	1.43
8	1.2	74	3.5	5.07	427324.00	2535.24	1.36
9	1.0	78	3.5	9.01	489852.33	3770.10	1.27
10	1.0	70	3.5	4.75	514094.66	2194.78	1.41
11	1.2	70	3.8	3.85	414681.33	1998.42	1.41
12	1.4	78	3.5	6.66	358455.33	3424.63	1.27
13	1.2	78	3.2	7.36	418862.00	3578.15	1.24
14	1.2	74	3.5	5.08	424021.33	2544.46	1.35
15	1.2	74	3.5	5.12	427172.33	2569.48	1.35
16	1.0	74	3.8	6.01	492396.66	2529.31	1.43
17	1.2	70	3.2	3.08	435635.66	2189.98	1.38

#### 3.2.1 Response Surface Analysis

The influential factors selected for optimization using the BBD were thoroughly evaluated through quadratic polynomial model fitting, which accounted for both main and interaction effects. The DoE software suggested 17 experimental runs, which were carried out and assessed for key responses: Rt (R1), peak area (R2), NTP (R3), and Tf (R4) as detailed in Table 3 and Table S4. The model’s F-values for Rt (247.80; p < 0.0001), peak area (765.50; p < 0.05), NTP (179.77; p < 0.05), and Tf (14.78; p < 0.0002) indicated statistical significance for all responses. The resulting quadratic equations for Rt, peak area, NTP, and Tf are presented as eqs 5–8, respectively.

5 $$Rt=5.10-0.8797A+1.95B+0.1471C-0.2368AB+0.0047AC-0.1408BC+0.2593A^{2}+0.5885B^{2}-0.2210C^{2}$$

6 $$\mathrm{Peak}\;\mathrm{area}=4.244E+05-68707.79A-9164.21B-11521.75+3713.25AB-138.67AC-1533.67BC+14108.29A^{2}-5940.54B^{2}-3215.46C^{2}$$

7 $$\mathrm{NTP}=2546.27-123.50A+694.44B-115.22C-50.72AB-1.74AC-42.15BC+31.56A^{2}+282.60B^{2}-82.73C^{2}$$

8 Tf=1.39+0.0006A−0.0783B+0.0929C

### 3.3 Effect of Independent Variables on Responses

#### 3.3.1 Effect on Retention Time (Rt)

The 2D contour and 3D response surface plots illustrated how the Rt of MGF lowered significantly with rising flow rate and declining buffer ratio, as evidenced by a quadratic model (p < 0.0001) (Figure 3). Increased flow rate (A) reduced the time spent by MGF in the column, leading to quicker elution through less interaction with the stationary phase. On the other hand, decreased phosphate buffer ratio (B) reduced ionic interaction and ion suppression, lowering MGF Rt in the column. Considering MGF’s polyhydroxy xanthonoid molecular structure and the strong elution strength of the mobile phase, the observed pattern is scientifically justified. Out of all the factors, the ratio of the buffer had a positive effect, while buffer pH had a negligible influence.^32^

**Figure 3:**
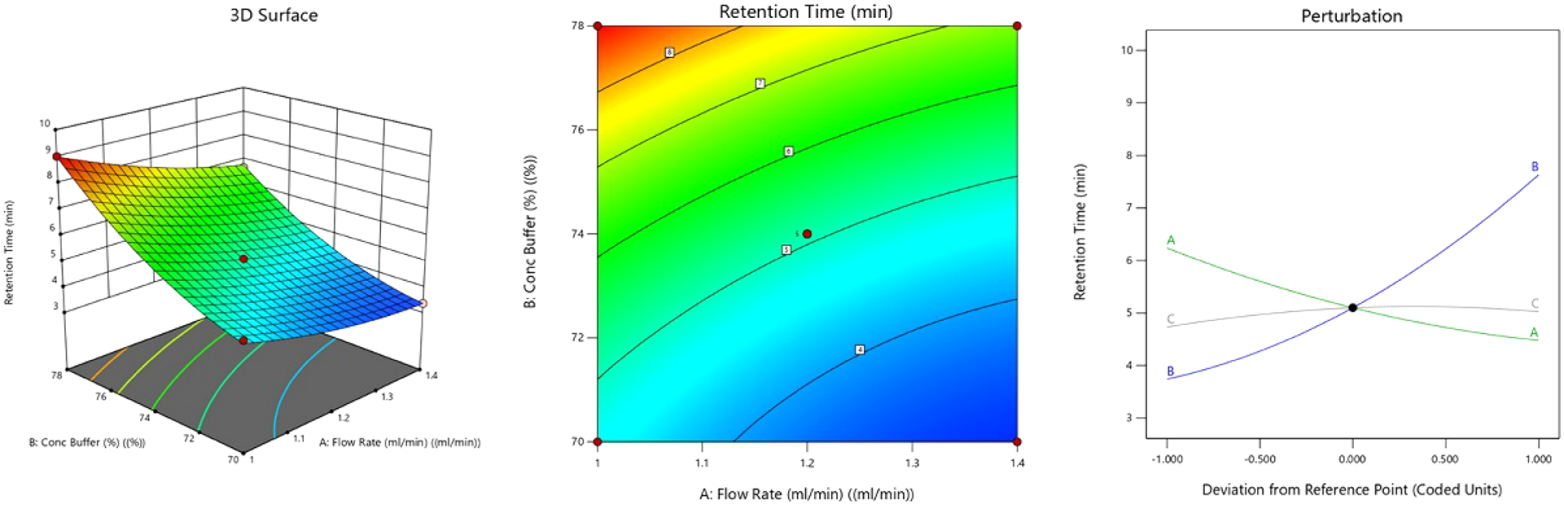
3D-response surface, 2D-contour plot, and perturbation plot graph representing the effect of CMPs on retention time.

#### 3.3.2 Effect on Peak Area

The fitted quadratic model was statistically acceptable with a significant overall model p-value (<0.05) and good correspondence between the actual and predicted responses, reflecting acceptable prediction of peak area behavior. Among the variables, flow rate (A) had the most notable negative linear effect on peak area, as evidenced by both the 3D surface and perturbation plots (Figure 4). Higher flow rates generally decrease the peak area due to reduced interaction time between the analyte and the stationary phase. The buffer ratio affects the elution strength and the separation efficiency, while Buffer pH is crucial for maintaining the stability and ionization state of MGF, which affects its interaction with the stationary phase. The buffer ratio (B) and buffer pH (C) also had a less intense negative effect, probably because of their impacts on the solubility of the analyte or detection response. The interaction between the variables AB showed a positive effect, which enhanced the peak area, whereas AC and BC showed a negative effect on the peak area.^18,33^

**Figure 4:**
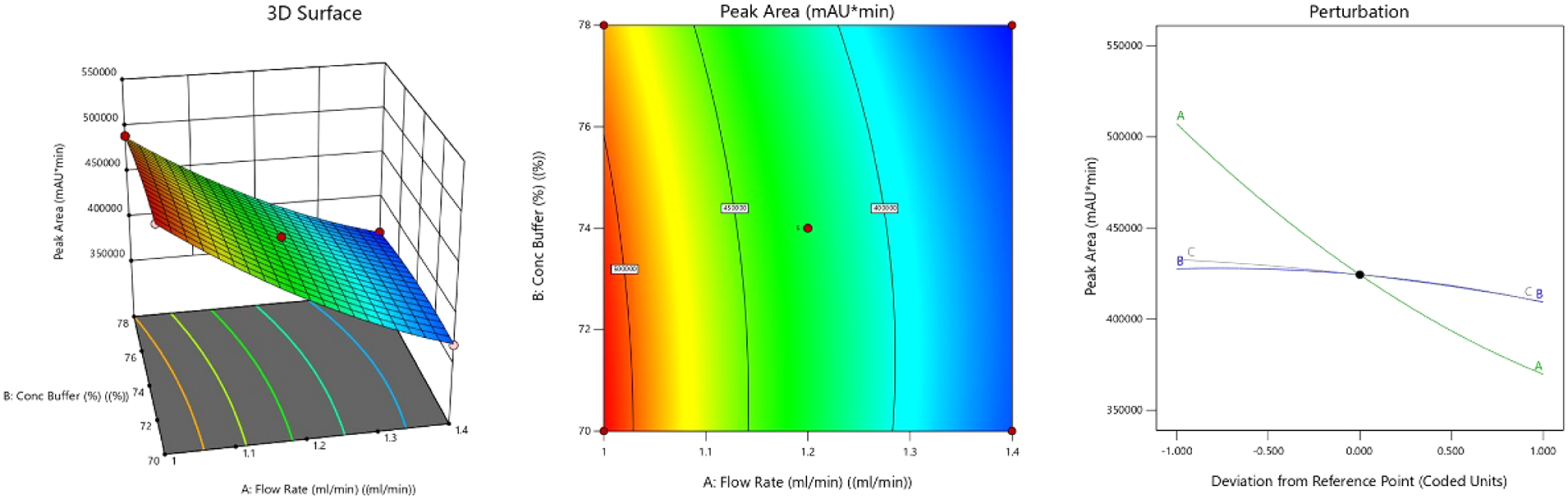
3D-response surface, 2D-contour plot, and perturbation plot graph representing the effect of CMPs on peak area.

#### 3.3.3 Effect on Number of Theoretical Plates

The quadratic model for NTP was statistically significant (p < 0.05), with all factors showing a significant effect on NTP (p < 0.05). The buffer ratio (B) had a positive influence on NTP, indicating enhanced column efficiency, resulting in an increase in NTP. In contrast, flow rate (A) and pH of the buffer (C) are inversely proportional to NTP, showing decreased efficiency at increased flow rates and Buffer pH (Figure 5). Quadratic terms of buffer ratio and pH illustrate a nonlinear relationship, pointing to the need for balanced conditions. Interaction terms like AB and BC also indicate that concurrent changes in flow rate with buffer ratio or pH can negatively impact efficiency.^34–37^

**Figure 5:**
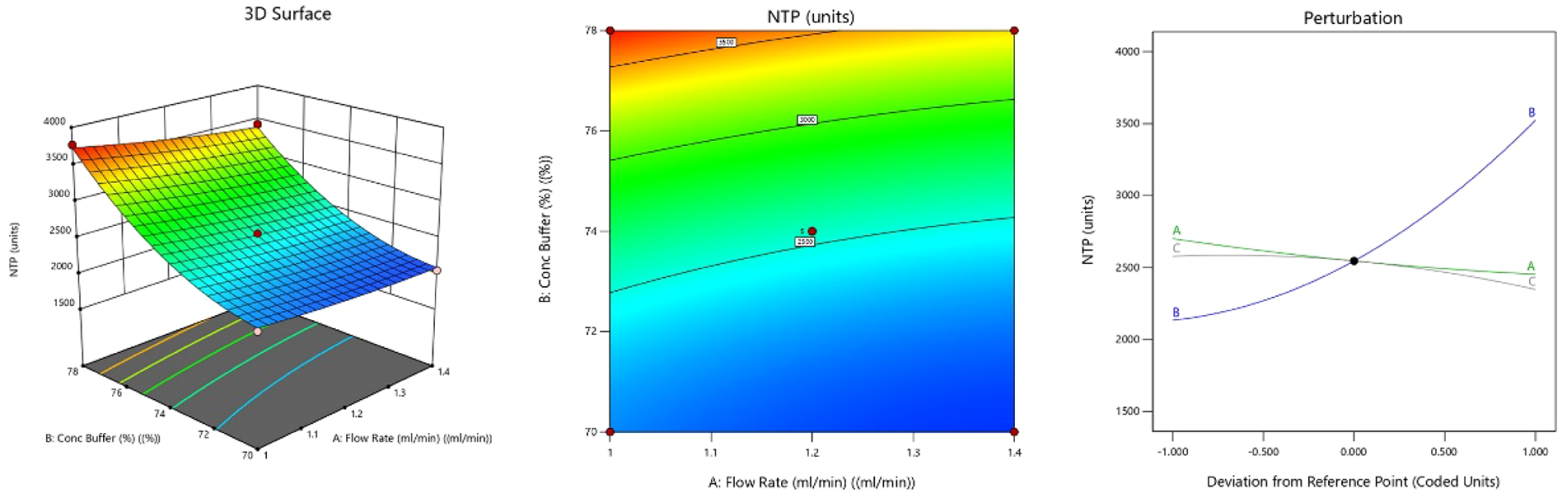
3D-response surface, 2D-contour plot, and perturbation plot graph representing the effect of CMPs on NTP.

#### 3.3.4 Effect on Tailing Factor (Tf)

MGF, a polyphenolic xanthonoid derivative with multiple hydroxy groups and intermediate polarity, requires proper regulation of pH, and buffer concentration is essential to reduce peak tailing.^38^ According to the regression model and surface plots, the Tf reduces significantly with a rising buffer ratio and increases at elevated pH (Figure 6). At low pH, MGF is predominantly in the un-ionized form, decreasing its electrostatic attraction with silanol groups of the stationary phase, thus enhancing peak symmetry.^39,40^ In addition, raising the phosphate buffer ratio increases ionic strength by suppressing undesirable secondary interactions, further decreasing peak tailing. The flow rate, though part of the model, demonstrates negligible influence on the Tf. Hence, to obtain maximum elution of MGF with a narrow, symmetrical peak, it is advisable to employ a phosphate buffer at a low pH and high buffer ratio in the mobile phase.^34^

**Figure 6:**
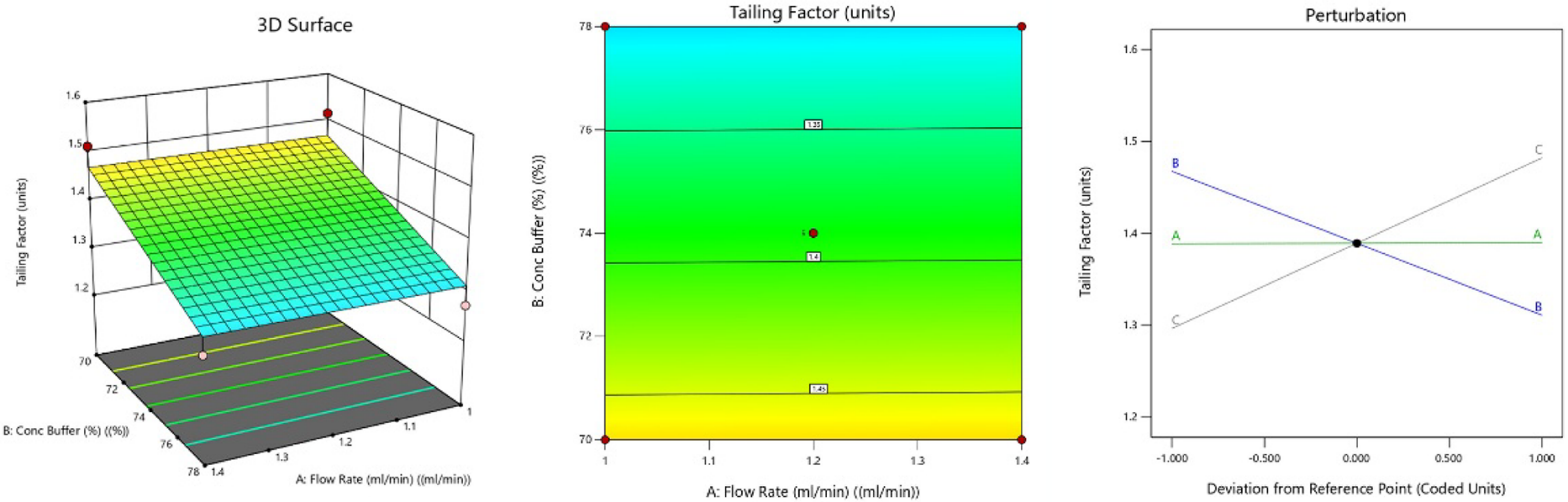
3D-response surface, 2D-contour plot, and perturbation plot graph representing the effect of CMPs on tailing factor.

### 3.4 Optimized Method from DoE Software Solution

The chromatographic parameters were refined using numerical optimization and graphical analysis to achieve optimal Rt, enhanced peak area, increased NTP, and reduced Tf as defined by CAAs. The optimal configuration, which yielded a desirability value of 0.965, comprised a mobile phase consisting of acetonitrile (30%) and methanol (70%) combined with a 10 mM phosphate buffer at pH 3.2 in a volume ratio of 22:78 v/v. The system operated at a 1.0 mL/min flow rate, with an injection volume set at 10 μL. The responses obtained under the specified chromatographic conditions were Rt of 8.36 min, peak area 498,335, and NTP of 3799.5 with a Tf of 1.2. The optimized method was validated through three replicate runs, demonstrating no statistically significant differences from the predicted values (p > 0.05). Additionally, graphical optimization using overlay plots (Figure 7) identified a continuous yellow region representing the design space, defined as the multivariate combination of method parameters within the studied ranges where all CAAs simultaneously meet predefined acceptance criteria. This model-predicted design space confirms method robustness in accordance with the QbD principles described in ICH Q14.

**Figure 7:**
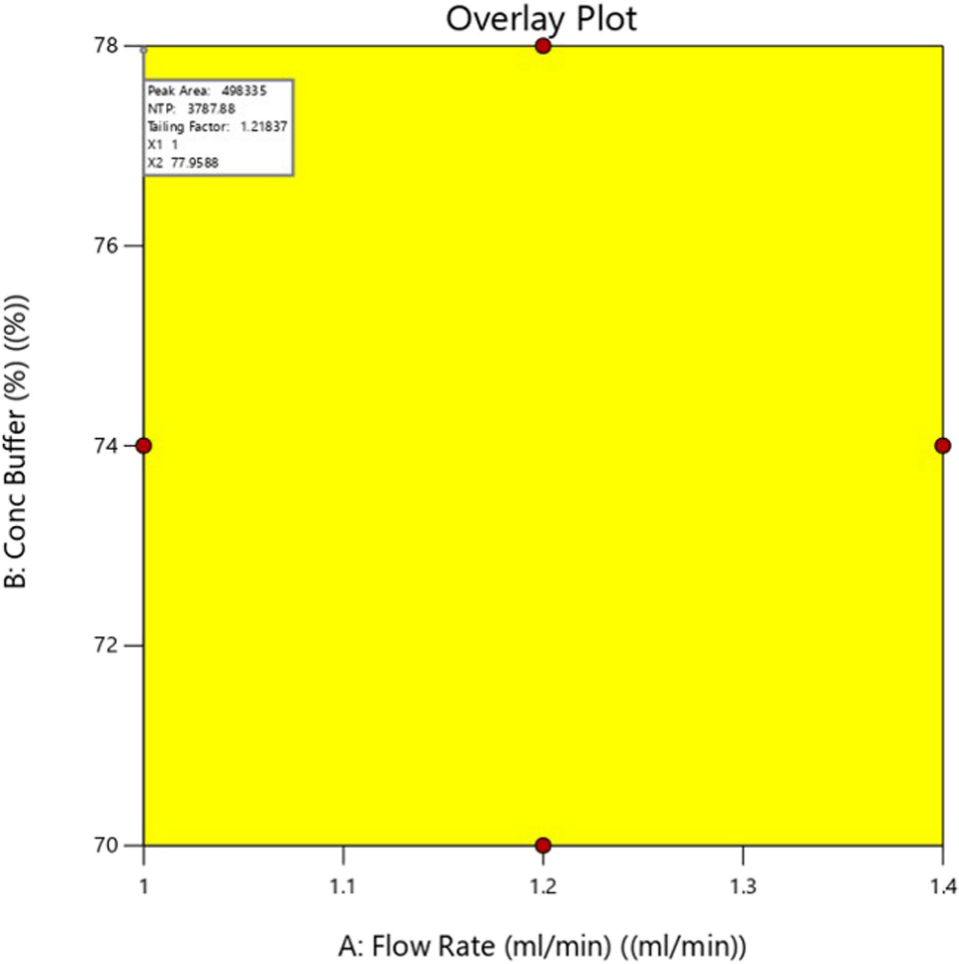
Overlay plot depicting the acceptable factors and responses

### 3.5 Validation of BBD Optimized Method

#### 3.5.1 System Suitability

System suitability testing was performed to ensure the integrated performance of the entire analytical system, including instrumentation, sample preparation, and chromatographic conditions (Figure S1). The developed method met all the system suitability criteria, with %RSD values for Rt and peak area maintained below 1%, confirming the method’s consistency and reproducibility. Detailed results are summarized in Table 4.

**Table 4:** Table Representing the Optimized Validation Parameters of MGF

HPLC validation parameters			
1. System suitability			
S. No.	Parameter	Average ± SD (n = 6)	% RSD
1	Analyte peak area (mV.min)	287865.80 ± 2139.40	0.74
2	Rt (min)	8.36 ± 0.008	0.10
3	NTP (per column length)	3768.03 ± 21.71	0.57
5	Tf (10%)	1.19 ± 0.008	0.72

2. Linearity	
Linearity range	50–5000 ng/mL
R^2^	0.999
Regression equation	y = 57.384x – 3008.6

3. LOD and LOQ	
LOD	1.43 ng/mL
LOQ	4.36 ng/mL

4. Precision						
	0 h		12 h		24 h	
Concentration (ng/mL)	%RSD-Rt	%RSD-Area	%RSD-Rt	%RSD-Area	%RSD-Rt	%RSD-Area
150	0.481	1.992	0.728	1.676	0.681	0.588
3000	0.649	0.742	0.157	0.767	0.731	0.334
4000	0.936	0.299	0.632	0.283	0.208	0.301

5. Accuracy					
S. No.	%Level	Spiked concentration (ng/mL)	Peak area (mV.min)	Recovered concentration (ng/mL)	%Recovery
1	75	1875	99389.33 ± 292.88	1793.80 ± 5.15	95.66 ± 0.27
2	100	2500	134826 ± 436.80	2417.46 ± 7.68	96.69 ± 0.30
3	125	3125	171177.33 ± 601.08	3057.23 ± 10.57	97.83 ± 0.33

6. Robustness						
		Levels	Retention time		%Recovery	
			Mean ± SD (n = 3)	%RSD	Mean ± SD (n = 3)	%RSD
1	Flow rate (mL)	0.9	9.11 ± 0.012	0.380	105.62 ± 0.31	0.300
		1.1	7.52 ± 0.016	0.215	100.41 ± 0.25	0.257
2	Mobile phase ratio (%)	76	6.66 ± 0.025	0.380	105.18 ± 1.68	1.600
		80	10.88 ± 0.091	0.844	109.38 ± 0.93	0.858
3	Wavelength (nm)	254	8.51 ± 0.009	0.113	107.67 ± 0.38	0.358
		260	8.52 ± 0.011	0.143	105.77 ± 0.335	0.336
4	Injection volume (μL)	8	9.11 ± 0.008	0.099	93.67 ± 0.16	0.180
		12	8.32 ± 0.021	0.257	108.82 ± 0.99	0.911
5	pH of mobile phase	2.7	8.43 ± 0.030	0.361	104.54 ± 1.79	1.717
		3.7	8.44 ± 0.030	0.356	107.97 ± 0.379	0.351

#### 3.5.2 Specificity

The Rt of MGF was recorded at 8.36 min. No interfering peaks were observed in the blank sample at the same Rt, indicating that the method is specific to MGF without interference from excipients or solvent peaks. Representative chromatograms of the blank and MGF standard are shown in Figure 8.

**Figure 8:**
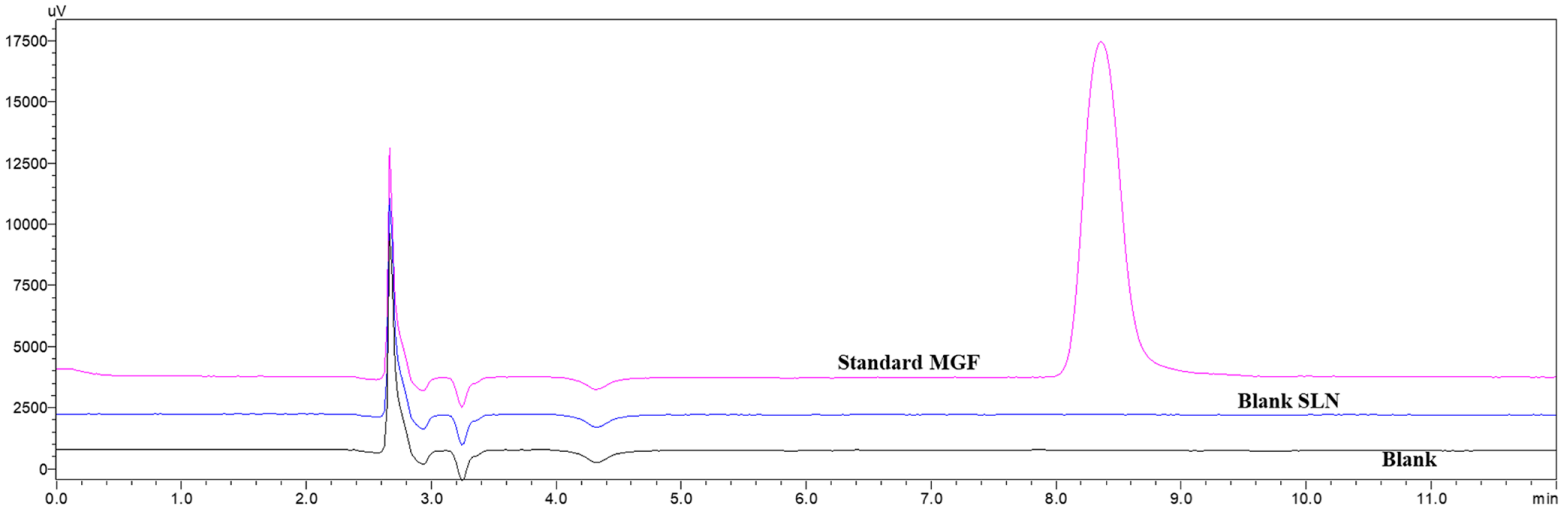
Chromatogram representing blank, blank SLN and MGF standard. Overlay chromatograms are displayed in μV; quantitative analyses were performed using absorbance units (mAU).

##### 3.5.2.1 Stability Studies

Forced degradation studies were carried out to evaluate the stability-indicating property of the developed HPLC method and to understand the degradation profile of the drug under different stress conditions. Forced degradation studies assist in the identification of possible degradation products and in determining whether the method can adequately separate the intact drug from its degradation products.^41^ When treated with 0.1 N of HCl for the analysis of acid hydrolysis, and 3% H_2_O_2_ for oxidation, no change was observed in the original peak area throughout the time period in all the conditions, such as 4 °C, 60 °C, and room temperature. Similarly, no degradation was found with 1 N HCl, under 4 °C and room temperature. While at 60 °C, the peak area was reduced over time, but not significantly, indicating that MGF is not susceptible to acid hydrolysis. Under photodegradation, when exposed to normal daylight, MGF slightly degraded. There was no change in peak area during thermal degradation for MGF when treated with 60 °C and kept in an incubator, which concludes that the drug was stable against the thermal stress.^32,42,43^

MGF was highly sensitive to alkali environments, where complete degradation was seen when treated with 0.1 N NaOH by the 8 h. However, by 2 and 4 h, two minor degradation peaks were observed in HPLC. The degradation mechanism primarily involves the cleavage of the tricyclic ring structure of MGF, leading to the formation of smaller molecular fragments. This process is facilitated by the strong alkaline nature of sodium hydroxide, which effectively breaks down the complex molecular structure of MGF.^32^ Similarly, with 1 N NaOH, a small, degraded peak was found at 4^th^ and 8^th^ hours in at 4 °C, whereas with 60 °C and room temperature, complete degradation was seen as soon as MGF came in contact with NaOH solution.^32,44^ The degradation behavior of MGF with different stressors is represented in Table S5 (Figure 9 and Figure S2).

**Figure 9:**
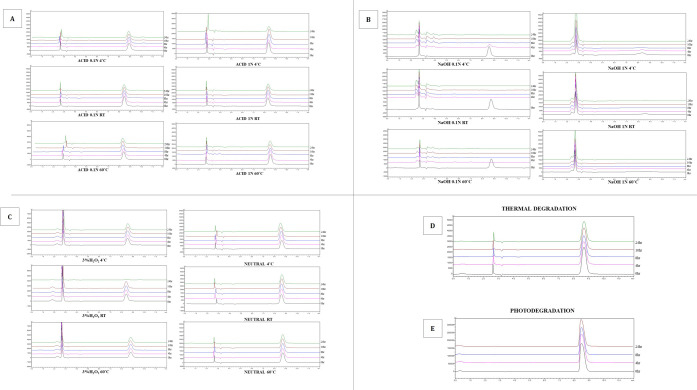
Overlay of HPLC chromatograms of Mangiferin when exposed to (A) 0.1 and 1.0 N of HCl, (B) 0.1 and 1.0 N of NaOH, (C) 3% H_2_O_2_ oxidation and neutral stress condition, (D) thermal degradation conditions and (E) photodegradation under different temperatures like 4 °C, RT and 60 °C.

#### 3.5.3 Linearity

Linearity was established by evaluating the detector’s response across various analyte concentrations. The calibration curve for MGF exhibited excellent linearity in the concentration range of 50–5000 ng/mL. The regression equation was y = 57.384x – 3008.6, with a correlation coefficient (R^2^) of 0.9999, indicating a strong linear relationship. The linearity graph is depicted in Figure S3.

#### 3.5.4 Accuracy

Accuracy refers to measuring how well the experimental result corresponds to the actual value. Accuracy was assessed by determining the percent recovery of MGF at three concentration levels: 75%, 100%, and 125% of the nominal concentration. The recoveries were within ± 3%, demonstrating the method’s accuracy in quantifying the analyte. These findings confirm that the sample preparation and analytical procedure can deliver accurate results (Table 4).

#### 3.5.5 Precision

A precision experiment measures the consistency (degree of variability) of repeated determinations of the same sample analyzed under the same analytical conditions. Precision was evaluated through intraday and interday repeatability studies. For intraday precision, %RSD values of peak areas at low, medium, and high concentrations were below 2%. Interday precision, assessed across two separate days, showed %RSD values below 2%. These results fall within the acceptable range per ICH Q2­(R2) guidelines, confirming that the method is precise and reproducible for routine analysis (Table 4 and Table S6).

#### 3.5.6 Limit of Detection (LOD) and Limit of Quantitation (LOQ)

The LOD and LOQ were determined to assess the sensitivity of the method. The LOD, indicating the lowest detectable concentration, was calculated to be 1.43 ng/mL. In contrast, the LOQ, representing the lowest quantifiable concentration with acceptable precision and accuracy, was found to be 4.36 ng/mL. The suitability of the estimated LOQ was verified by analyzing samples at this concentration using the developed method, in which a reproducible, well-defined peak was obtained. These results demonstrate the high sensitivity of the proposed RP-HPLC method for the detection and quantification of MGF.

#### 3.5.7 Robustness

The robustness of the method was evaluated by introducing minor variations in chromatographic parameters, such as flow rate, mobile phase composition, and other parameters. The %RSD for peak area and Tf remained below 2%, indicating that the method is robust and unaffected by minor procedural deviations (Table 4).

### 3.6 Application of Optimized HPLC Method

#### 3.6.1 Drug Content MGF-SLNs

Mangiferin-loaded SLNs had a drug content of 84.45 ± 0.21%.

#### 3.6.2 In Vitro Drug Release Studies

In vitro drug release of MGF-SLN was carried out by dialysis bag technique where, pure MGF showed poor and irregular release pattern, with an optimal cumulative drug release (%CDR) of about 41% at 10 h, after which it gradually decreased, due to the poor aqueous solubility and possible degradation/precipitation upon extended exposure. In contrast, MGF-SLNs exhibited sustained release of up to 81% in 72 h (Figure 10). The higher release from the formulation is due to better solubilization, nanosizing, and matrix-based diffusion from the solid lipid nanoparticle system. Furthermore, release kinetics were applied using different best-fit kinetic models. The MGF lipid nanoparticles exhibited a good fit to the Higuchi model (R^2^ = 0.8447), consistent with a diffusion-controlled mechanism, as also seen for sustained release.^16,45,46^ These findings support that lipid-based nanoformulation significantly increases release efficiency and extends the drug availability of MGF.

**Figure 10:**
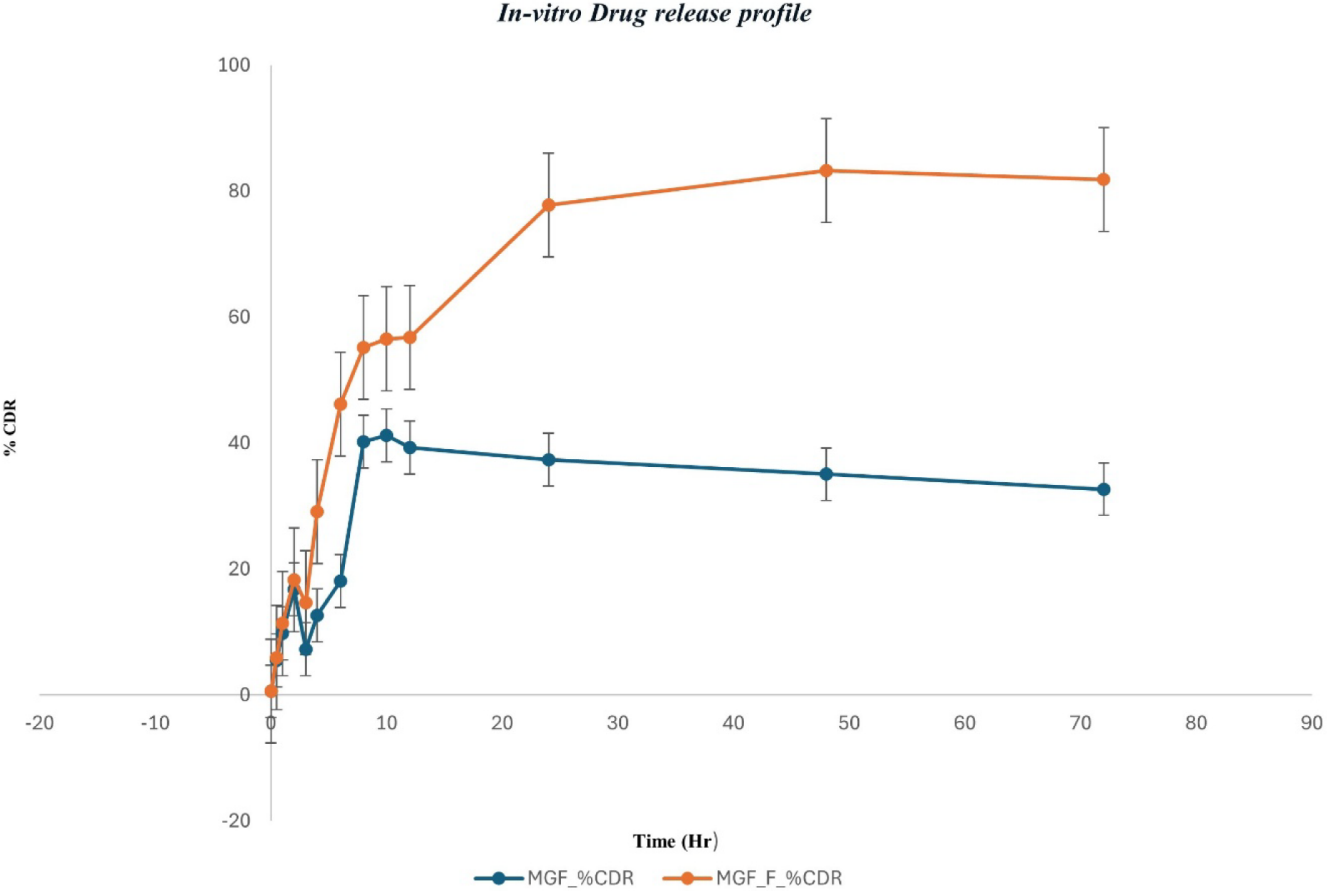
In vitro release studies of MGF and MGF-SLNs.

## 4 Conclusion

The current research effectively developed a scientifically valid and robust HPLC procedure for quantitatively determining MGF using an AQbD approach. Method screening was initially accomplished through the Taguchi OA design, which identified critical method parameters. BBD was then performed to optimize the chromatographic conditions for the peak shape, mobile phase ratio, retention time, and other parameters. The developed method was validated in compliance with ICH Q2­(R2) guidelines and demonstrated excellent specificity, linearity, accuracy, precision, and robustness. The developed method is simple with isocratic flow, cost-efficient, and requires minimal organic solvent. Validation parameters demonstrated high sensitivity at the nanogram level and confirmed excellent robustness.

The validated method was effectively applied to estimate drug content and evaluate in vitro drug release kinetics from MGF-SLNs, confirming its utility in formulation analysis. Additionally, forced degradation studies under several stress conditions (acidic, basic, oxidative, thermal, and photolytic) validated the stability-indicating potential of the method, as it was able to differentiate MGF from its degradation products significantly. Overall, the proposed method is of great analytical utility for quality control, formulation, and stability testing of MGF and its nanoformulations.

## Supplementary Material



## Data Availability

Data used are available throughout the manuscript text.

## Notes

Method variables for Taguchi OA design; runs from Taguchi OA design; CMPs and levels by Box–Behnken design; statistical model parameters of BBD–DoE results; forced degradation data of MGF; system suitability chromatograms; overlay of HPLC chromatograms of MGF with stressors; calibration curve; precision data of MGF (PDF)

## Notes

A.P.K.: Writing—review and editing, Writing—original draft, Software, Methodology, Investigation, Formal analysis. L.K.: Writing—review and editing, Validation, Supervision, Formal analysis, Project administration. S.M.: Writing—review and editing, Validation, Supervision, Project administration. P.G.N.: Writing—review and editing, Conceptualization, Visualization, Supervision, Review and editing. K.N.: Writing—review and editing, Validation, Supervision, Methodology, Conceptualization

## References

[1] Adamu A., Li S., Gao F., Xue G. (2024). The Role of Neuroinflammation in
Neurodegenerative Diseases: Current Understanding and Future Therapeutic
Targets. Front. Aging Neurosci..

[2] Houldsworth A. (2023). Role of Oxidative
Stress in Neurodegenerative Disorders: A Review of Reactive Oxygen
Species and Prevention by Antioxidants. Brain
Commun..

[3] Venkatesan R., Ji E., Kim S. Y. (2015). Phytochemicals That Regulate Neurodegenerative Disease
by Targeting Neurotrophins: A Comprehensive Review. Biomed. Res. Int..

[4] Piccialli I., Tedeschi V., Caputo L., D’Errico S., Ciccone R., De Feo V., Secondo A., Pannaccione A. (2022). Exploring
the Therapeutic Potential of Phytochemicals in Alzheimer’s
Disease: Focus on Polyphenols and Monoterpenes. Front. Pharmacol..

[5] Singh S. K., Kumar Y., Kumar S. S., Sharma V. K., Dua K., Samad A. (2009). Antimicrobial Evaluation
Of Mangiferin Analogues. Indian J. Pharm. Sci..

[6] Núñez
Sellés A. J., Vélez Castro H.
T., Agüero-Agüero J., González-González J., Naddeo F., De Simone F., Rastrelli L. (2002). Isolation and Quantitative Analysis of Phenolic Antioxidants,
Free Sugars, and Polyols from Mango (Mangifera Indica L.) Stem Bark
Aqueous Decoction Used in Cuba as a Nutritional Supplement. J. Agric. Food Chem..

[7] Bairy I., Reeja S., Siddharth, Rao P. S., Bhat M., Shivananda P. G. (2002). Evaluation
Of Antibacterial Activity Of Mangifera, Indica On Anaerpbic Dental
Microglora Based On In Vivo Studies. Indian
J. Pathol. Microbiol..

[8] Du S., Liu H., Lei T., Xie X., Wang H., He X., Tong R., Wang Y. (2018). Mangiferin: An Effective Therapeutic
Agent against Several Disorders (Review). Mol.
Med. Rep..

[9] Gururaja G. M., Mundkinajeddu D., Kumar A. S., Dethe S. M., Allan J. J., Agarwal A. (2017). Evaluation of Cholesterol-Lowering Activity of Standardized
Extract of Mangifera Indica in Albino Wistar Rats. Pharmacogn. Res..

[10] Jiang T., Han F., Gao G., Liu M. (2020). Mangiferin Exert Cardioprotective
and Anti-Apoptotic Effects in Heart Failure Induced Rats. Life Sci.

[11] Qin Z., Wang S., Lin Y., Zhao Y., Yang S., Song J., Xie T., Tian J., Wu S., Du G. (2018). Antihyperuricemic Effect
of Mangiferin Aglycon Derivative J99745
by Inhibiting Xanthine Oxidase Activity and Urate Transporter 1 Expression
in Mice. Acta Pharm. Sin. B.

[12] Peng S., Hou Y., Yao J., Fang J. (2019). Neuroprotection of Mangiferin against
Oxidative Damage via Arousing Nrf2 Signaling Pathway in PC12 Cells. BioFactors.

[13] Saha S., Sadhukhan P., Sil P. C. (2016). Mangiferin: A Xanthonoid with Multipotent
Anti-Inflammatory Potential. BioFactors.

[14] Noratto G.
D., Bertoldi M. C., Krenek K., Talcott S. T., Stringheta P. C., Mertens-Talcott S. U. (2010). Anticarcinogenic Effects of Polyphenolics from Mango
(Mangifera Indica) Varieties. J. Agric. Food
Chem..

[15] Gottlieb M., Leal-Campanario R., Campos-Esparza M. R., Sánchez-Gómez M. V., Alberdi E., Arranz A., Delgado-García J. M., Gruart A., Matute C. (2006). Neuroprotection by Two Polyphenols
Following Excitotoxicity and Experimental Ischemia. Neurobiol. Dis..

[16] Adin S. N., Gupta I., Rashid M. A., Alhamhoom Y., Aqil M., Mujeeb M. (2023). Nanotransethosomes
for Enhanced Transdermal
Delivery of Mangiferin against Rheumatoid Arthritis: Formulation,
Characterization, Invivo Pharmacokinetic and Pharmacodynamic Evaluation. Drug Delivery.

[17] Santonocito D., Vivero-Lopez M., Lauro M. R., Torrisi C., Castelli F., Sarpietro M. G., Puglia C. (2022). Design of Nanotechnological Carriers
for Ocular Delivery of Mangiferin: Preformulation Study. Molecules.

[18] Naveen P., Lingaraju H. B., Prasad K. S. (2017). Rapid Development
and Validation
of Improved Reversed-Phase High-Performance Liquid Chromatography
Method for the Quantification of Mangiferin, a Polyphenol Xanthone
Glycoside in Mangifera Indica. Pharmacogn. Res..

[19] Bonde S., Bonde C. G., Prabhakar B. (2019). Quality by Design Based Development
and Validation of HPLC Method for Simultaneous Estimation of Paclitaxel
and Vinorelbine Tartrate in Dual Drug Loaded Liposomes. Microchem. J..

[20] Prajapati P., Tamboli J., Surati P., Mishra A. (2021). Risk Assessment-Based
Enhanced Analytical Quality-by-Design Approach to Eco-Friendly and
Economical Multicomponent Spectrophotometric Methods for Simultaneous
Estimation of Montelukast Sodium and Bilastine. J. AOAC Int..

[21] Saini S., Sharma T., Patel A., Kaur R., Tripathi S. K., Katare O. P., Singh B. (2020). QbD-Steered
Development and Validation
of an RP-HPLC Method for Quantification of Ferulic Acid: Rational
Application of Chemometric Tools. J. Chromatogr.
B.

[22] Krishna M. V., Dash R. N., Jalachandra
Reddy B., Venugopal P., Sandeep P., Madhavi G. (2016). Quality by
Design (QbD) Approach
to Develop HPLC Method for Eberconazole Nitrate: Application Oxidative
and Photolytic Degradation Kinetics. J. Saudi
Chem. Soc..

[23] Li K., Zhang Y., Cao Y., Shi Y. (2010). Determination of chlorogenic
acid and mangiferin in Folium Pyrrosiae from different habitats and
species by HPLC. Zhongguo ZhongYao ZaZhi.

[24] Jain V., Paskanti Y. (2020). Department of Quality
Assurance, Oriental College of
Pharmacy, Navi Mumbai, Maharashtra, India. Novel HPLC Method for Simultaneous
Estimation of Gallic Acid, Protocatechuic Acid and Mangiferin in Mango. J. Sci. Innovative Res..

[25] Gil E. C., Hernández R. G., Paz L. N., Hoogmartens J. (2014). Determination
Of Mangiferin In Mangifera Indica L. Stem Bark Extract (vimang®)
And Pharmaceuticals By Liquid Chromatography. Emirates J. Food Agric..

[26] Nahata A., Sethiya N. K., Jain N., Dixit V. K. (2019). Analysis
of Scopoletin
and Mangiferin in Botanicals and Formulations of Shankhpushpi by HPLC. Herba Pol..

[27] Kammalla A. K., Ramasamy M. K., Aruna A., Kaliappan I. (2014). Development
and Validation of a RP-HPLC Method for the Simultaneous Determination
of Mangiferin, Ellagic Acid and Hydroxycitric Acid in Polyherbal Formulation. Pharmacogn. J..

[28] ICH Harmonised Guideline Analytical Procedure Development Q14; 2022. https://database.ich.org/sites/default/files/ICH_Q14_Document_Step2_Guideline_2022_0324.pdf. accessed 27 June 2025.

[29] ICH Harmonised for Better Health ICH-Guideline-Q2r2-Validation-Analytical-Procedures; 2022. https://www.ema.europa.eu/en/documents/scientific-guideline/ich-guideline-q2r2-validation-analytical-procedures-step-2b_en.pdf. accessed 27 June 2025.

[30] Abraham, J. International Conference On Harmonisation Of Technical Requirements For Registration Of Pharmaceuticals For Human Use. In Handbook of Transnational Economic Governance Regimes, Tietje, C. ; Brouder, A. , Eds.; Brill Nijhoff, 2010; pp. 1041–1053. DOI: 10.1163/ej.9789004163300.i-1081.897.

[31] Ahmad N., Khan M. F., Ullah Z., Chaudhary A. A., Alawam A. S., Khalid M. S., Ali M. T. (2024). Development
and
Evaluation of Polysorbate-80 Coated Mangiferin PLGA Nanoparticles
Used in the Treatment of Cerebral Ischemia. Polym. Bull..

[32] Khurana R. K., Kaur S., Kaur J., Singh B. (2017). Elucidation
of Stress-Induced
Degradation Products of Mangiferin: Method Development and Validation. Biomed. Chromatogr..

[33] Agrafiotou P., Maliakas C., Pappa-Louisi A., Sotiropoulos S. (2003). A General
Approach to the Derivation of Peak Area Flow Dependence in FIA and
HPLC Amperometric Detection. Electrochim. Acta.

[34] Lin S.-H., Chang K.-C., Chen Y.-C., Yu T., Pai S.-C. (2009). Examination
of the Temporal Effect in a Flow Injection Analysis System Using Multi-Channel
Absorbance Detection. J. Chromatogr. A.

[35] Pan L., LoBrutto R., Kazakevich Y. V., Thompson R. (2004). Influence of Inorganic
Mobile Phase Additives on the Retention, Efficiency and Peak Symmetry
of Protonated Basic Compounds in Reversed-Phase Liquid Chromatography. J. Chromatogr. A.

[36] Soriano-Meseguer S., Fuguet E., Port A., Rosés M. (2019). Influence
of the Acid-Base Ionization of Drugs in Their Retention in Reversed-Phase
Liquid Chromatography. Anal. Chim. Acta.

[37] Subirats X., Bosch E., Rosés M. (2004). Retention
of Ionisable Compounds
on High-Performance Liquid Chromatography: XV. Estimation of the pH
Variation of Aqueous Buffers with the Change of the Acetonitrile Fraction
of the Mobile Phase. J. Chromatogr. A.

[38] Kalíková K., Boublík M., Kučerová G., Kozlík P. (2018). The effect
of buffer concentration and cation type in the mobile phase on retention
of amino acids and dipeptides in hydrophilic interaction liquid chromatography. Chem. Pap..

[39] Mendoza-Sarmiento G., Rojas-Hernández A., Galano A., Gutiérrez A. (2016). A Combined
Experimental–Theoretical Study of the Acid–Base Behavior
of Mangiferin: Implications for Its Antioxidant Activity. RSC Adv..

[40] Smith E. A., Wirth M. J. (2004). pH Dependence of
Tailing in Reversed-Phase Chromatography
of a Cationic Dye: Measurement of the Strong Adsorption Site Surface
Density. J. Chromatogr. A.

[41] Blessy M., Patel R. D., Prajapati P. N., Agrawal Y. K. (2014). Development of Forced
Degradation and Stability Indicating Studies of Drugs—A Review. J. Pharm. Anal..

[42] Mokal R. R., Jadhav A. P. (2021). Forced Degradation
Studies of Mangiferin and Berberine
by High-Performance Thin-Layer Chromatography. JPC-J. Planar Chromatogr--Mod. TLC.

[43] Adin S. N., Gupta I., Aqil M., Mujeeb M., Najmi A. K. (2023). Application
of a Quality by Design-Based Approach in Development and Validation
of an RP-HPLC Method for Simultaneous Estimation of Methotrexate and
Mangiferin in Dual Drug-Loaded Liposomes. Biomed.
Chromatogr..

[44] Padh H., Parmar S., Patel B. (2017). Stability
Indicating Hptlc Method
For Estimation Of Mangiferin In Bulk And Dosage Form. Int. J. Pharm. Biol. Sci..

[45] Samadarsi R., Dutta D. (2020). Anti-Oxidative Effect
of Mangiferin-Chitosan Nanoparticles on Oxidative
Stress-Induced Renal Cells. Int. J. Biol. Macromol..

[46] Shaikenov R., Klimshina V., Generalova Y., Serbun P., Kosova A., Dorogov M., Morozkina S., Snetkov P. (2024). Electrospun Hyaluronan-Based
Nanofibers with Mangiferin: Preparation, Morphology, and Drug Release
Kinetics. Eng. Proc..

