# Supplementary material for: Reverse Phase High Performance Liquid Chromatography Method for the Estimation of Mangiferin in Nano Formulations: Development and Optimization by Analytical Quality-by-Design
Source: ACS Omega. 2026 Mar 14;11(11):18067–79. doi: 10.1021/acsomega.5c12935 (PMC13019371; doi:10.1021/acsomega.5c12935)
Supplement: Supplementary file 1 [file ao5c12935_si_001.pdf]

Supplementary file:

Reverse Phase High Performance Liquid Chromatography Method for the Estimation of Mangiferin in Nano Formulations: Development and Optimization by Analytical Quality-by-Design

Akshatha P Kamath[a], Lalit Kumar[b], Srinivas Mutalik[c], Krishnadas Nandakumar[a*], Pawan Ganesh Nayak[a*]

[a] Department of Pharmacology, Manipal College of Pharmaceutical Sciences, Manipal Academy of Higher Education (MAHE), Manipal 576104, Karnataka, India

[b] Department of Pharmaceutics, National Institute of Pharmaceutical Education & Research, Hajipur 844 102, Bihar, India

[c] Department of Pharmaceutics, Manipal College of Pharmaceutical Sciences, Manipal Academy of Higher Education (MAHE), Manipal 576104, Karnataka, India

Table s1: List of method variables for Taguchi OA design

	**Method Variables**	**Units**	**Low**	**High**
A	**Flow rate**	mL/min	0.9	1.2
B	**Injection Vol.**	µL	10	15
C	**Wavelength**	nm	256	260
D	**Oven temp.**	˚C	22	25
E	**Buffer ratio**	%	74	80
F	**Buffer strength**	mM	10	15
G	**Buffer pH**	units	3.0	4.0

Table s2: Experimental runs from Taguchi OA design

Run	Flow Rate (ml/min)	Inj. Vol (µL)	Wavelength (nm)	Oven Temp (˚C)	Buffer Ratio (%)	Buffer Strength (mM)	Buffer pH
1	1.2	10	260	22	80	10	4
2	0.9	10	256	22	74	10	3
3	0.9	10	256	25	80	15	4
4	1.2	15	256	25	74	10	4
5	1.2	10	260	25	74	15	3
6	1.2	15	256	22	80	15	3
7	0.9	15	260	25	80	10	3
8	0.9	15	260	22	74	15	4

Table s3: Critical method parameters and levels by Box-Behnken Design

Critical method parameters		Levels	
		Low	High
		(+1)	(-1)
Flow rate	(µL)	1	1.4
Concentration of Buffer	(%)	70	78
Buffer pH	(units)	3.2	3.8

Table s4: BBD–DoE results showing statistical model parameters (R^2, adjusted R^2, predicted R^2, %CV, standard deviation, and precision) for Retention Time, Peak Area, NTP, and Tailing Factor.

Retention Time		Peak Area		NTP		Tailing Factor	
**R^2**	0.9969	**R^2**	0.9990	**R^2**	0.9957	**R^2**	0.8533
**Adjusted R^2**	0.9928	**Adjusted R^2**	0.9977	**Adjusted R^2**	0.9902	**Adjusted R^2**	0.8194
**Predicted R^2**	0.9505	**Predicted R^2**	0.9947	**Predicted R^2**	0.9334	**Predicted R^2**	0.7140

**% CV**	2.45	**% CV**	0.5685	**% CV**	1.98	**% CV**	2.85
**Std. Dev.**	0.1321	**Std. Dev.**	2425.87	**Std. Dev.**	52.55	**Std. Dev.**	0.0394
**Precession**	57.026	**Precession**	86.24	**Precession**	43.435	**Precession**	17.892

Table s5: Forced degradation data of Mangiferin

Stressor	Conc. of stressor	Exposure temp.	Time period (hr)	Rt	Degradation peaks	% degradation	Remarks
**Acid Hydrolysis**	**0.1N HCl**	4˚C	24	8.24±0.15	No	-	Main peak remained intact. No degradation
		RT	24	8.51±0.01	No	-	
		60˚C	24	8.71±0.02	No	-	
	**1N HCl**	4˚C	24	8.34±0.02	No	-	Main peak remained intact. No degradation
		RT	24	8.34±0.02	No	-	
		60˚C	24	8.57±0.003	No	At 24hr 40%	The main peak area is reduced.
**Alkali Hydrolysis**	**0.1N NaOH**	4˚C	4	8.56±0.04	No	At 8hr 100%	80% degradation at 4th h. Complete degradation at 8th h.
		RT	4	-	5.6 & 6.9	At 8hr 100%	The main peak degraded into two small peaks, leading to 100% degradation.
		60˚C	4	-	5.6 & 6.9	At 8hr 100%	
	**1N NaOH**	4˚C		-	No	At 4hr 100%	Reduction in peak area leading to 100% degradation at 8th h
		RT		-	No	100%	Complete degradation of the drug as soon as it is added to the stressor.
		60˚C		-	No	100%	
**Oxidation**	**3% H_2O_2**	4˚C		8.5±0.01	No	-	The principal peak remained unchanged, indicating no degradation.
		RT		8.31±0.01	No	-	
		60˚C		8.79±0.02	No	-	
**Neutral Hydrolysis**	**MQ water**	4˚C		8.53±0.03	No	-	The principal peak remained unchanged, indicating no degradation.
		RT		8.66±0.03	No	-	
		60˚C		8.74±0.03	No	-	

**Thermal Degradation**	**80˚C**	80˚C		8.69±0.006	No	-	The principal peak remained unchanged, indicating no degradation.
**Photo degradation**	**Day light**	-		8.54±0.02	No	-	Main peak remained intact. No degradation

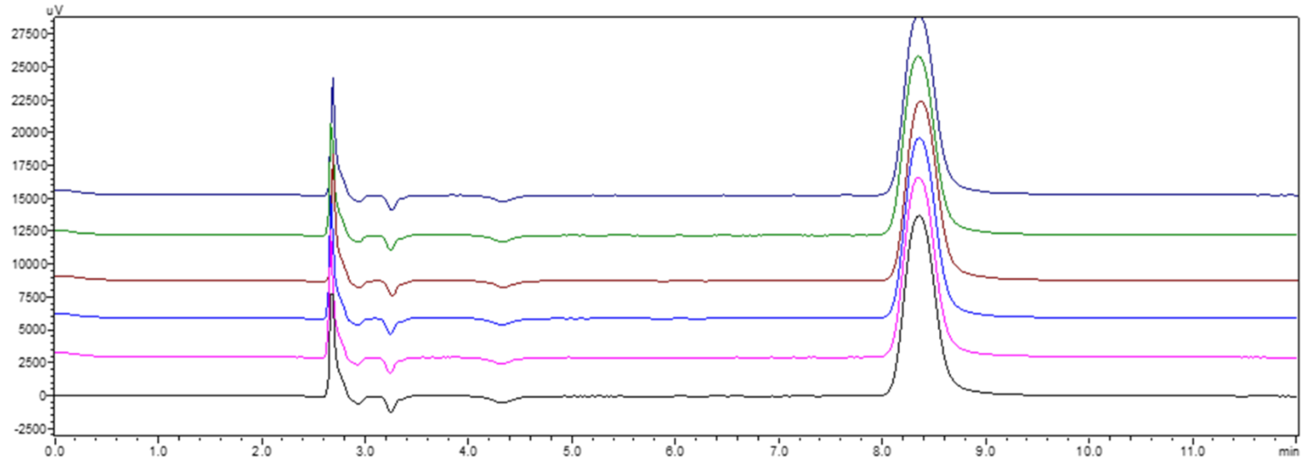

Figure s1: Chromatograms representing System suitability of MGF (n=6).

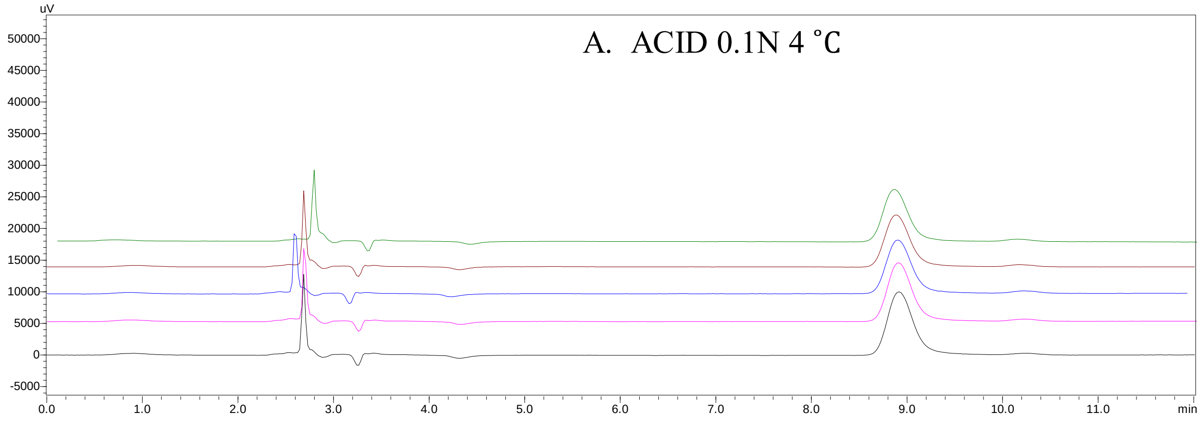

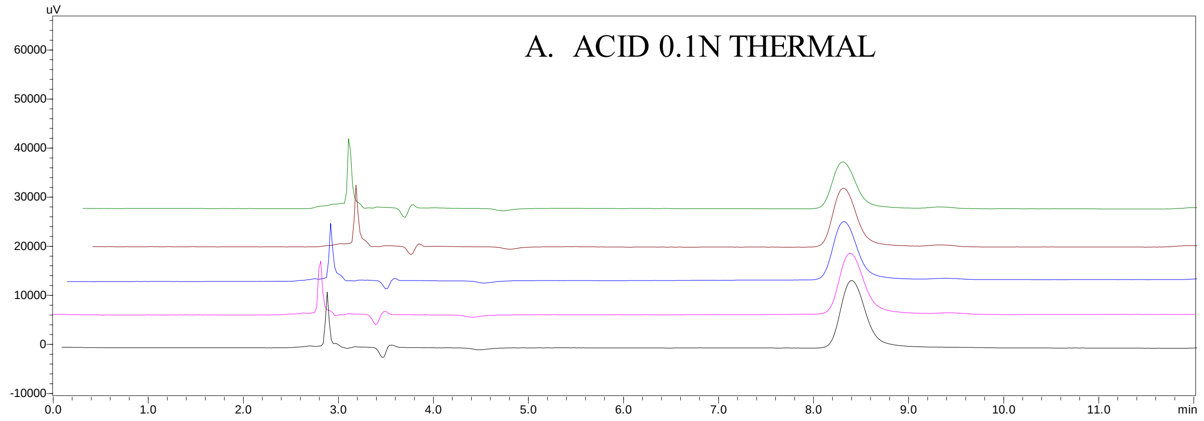
A. ACID 0.1N THERMAL
uV
60000
50000
40000
30000
20000
10000
0
-10000
0.0
1.0
2.0
3.0
4.0
5.0
6.0
7.0
8.0
9.0
10.0
11.0
min

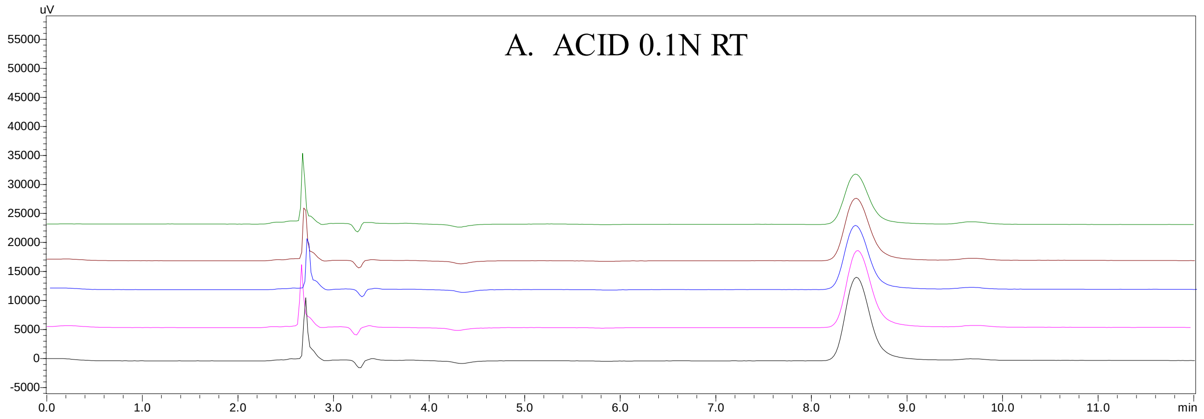
A. ACID 0.1N RT
uV
55000
50000
45000
40000
35000
30000
25000
20000
15000
10000
5000
0
-5000
0.0
1.0
2.0
3.0
4.0
5.0
6.0
7.0
8.0
9.0
10.0
11.0
min

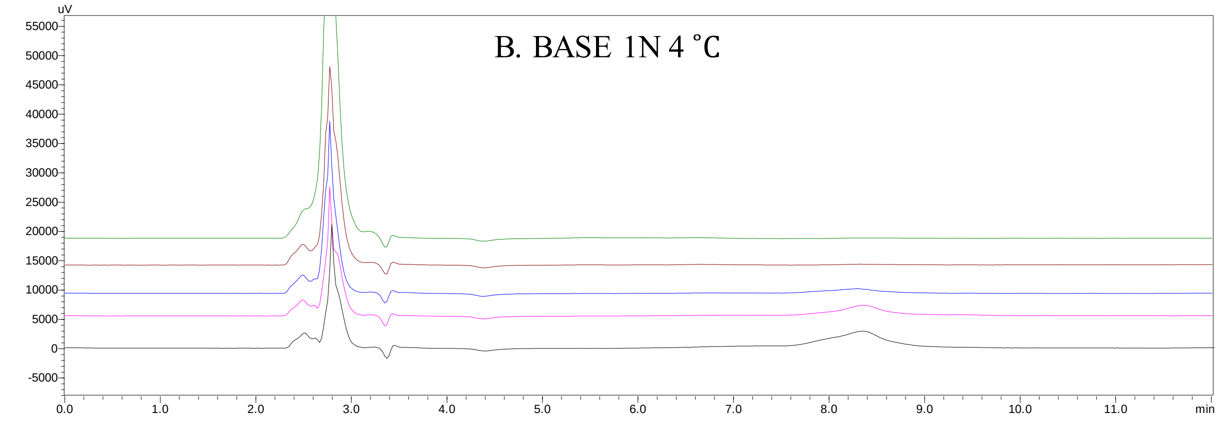
B. BASE 1N 4 ˚C
uV
55000
50000
45000
40000
35000
30000
25000
20000
15000
10000
5000
0
-5000
0.0
1.0
2.0
3.0
4.0
5.0
6.0
7.0
8.0
9.0
10.0
11.0
min

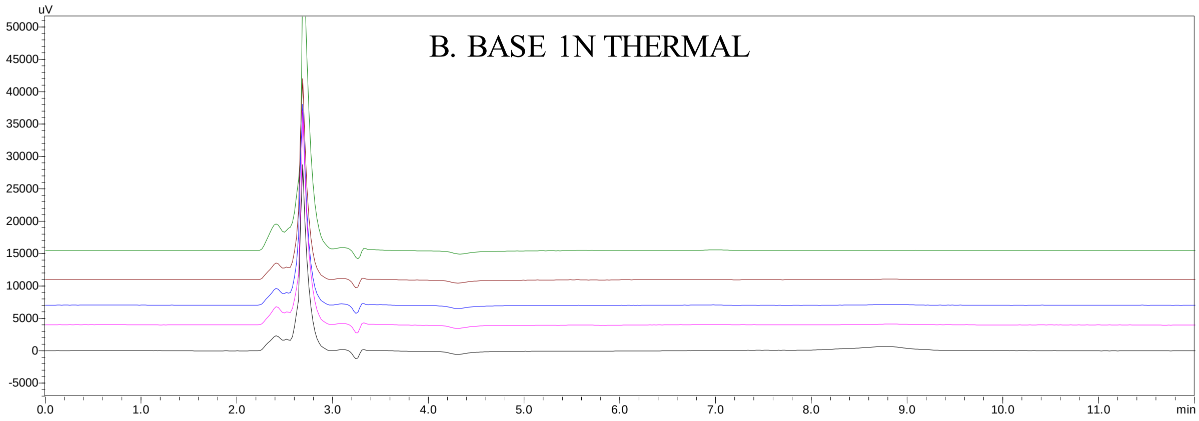
uV
B. BASE 1N THERMAL
min

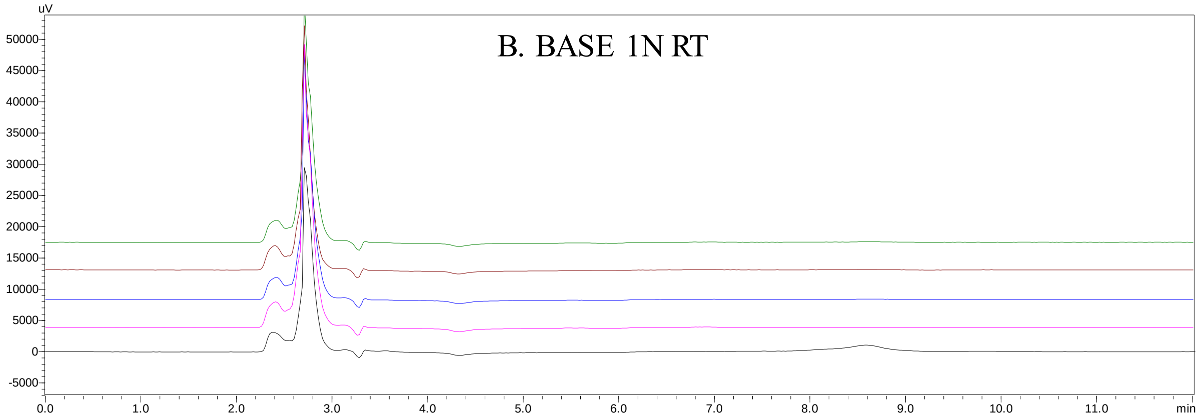
uV
B. BASE 1N RT
min

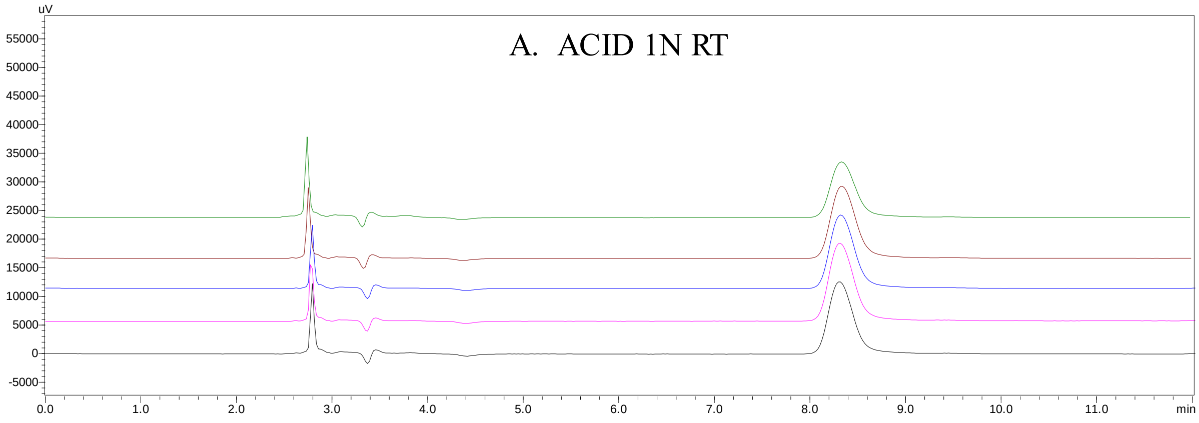
uV
A. ACID 1N RT
min

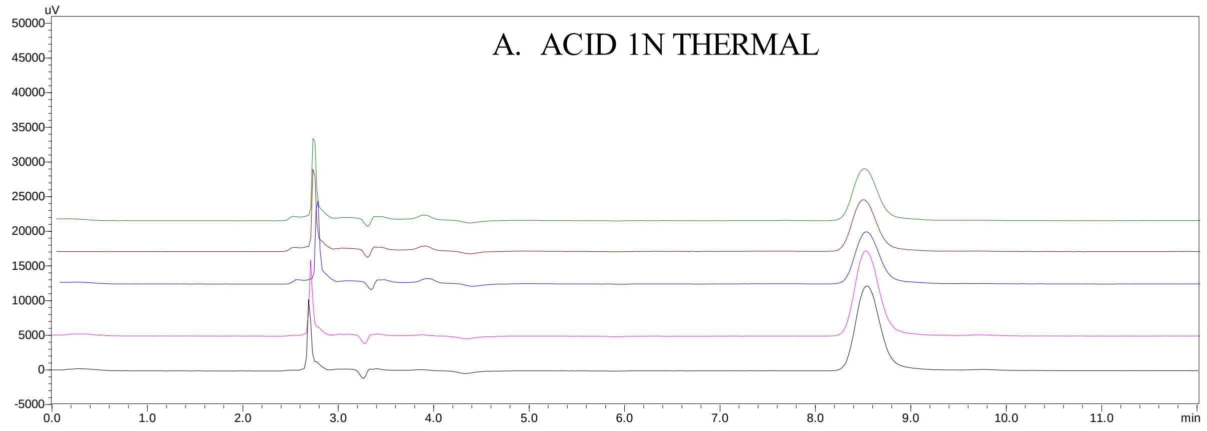
A. ACID 1N THERMAL
uV
50000
45000
40000
35000
30000
25000
20000
15000
10000
5000
0
-5000
0.0
1.0
2.0
3.0
4.0
5.0
6.0
7.0
8.0
9.0
10.0
11.0
min

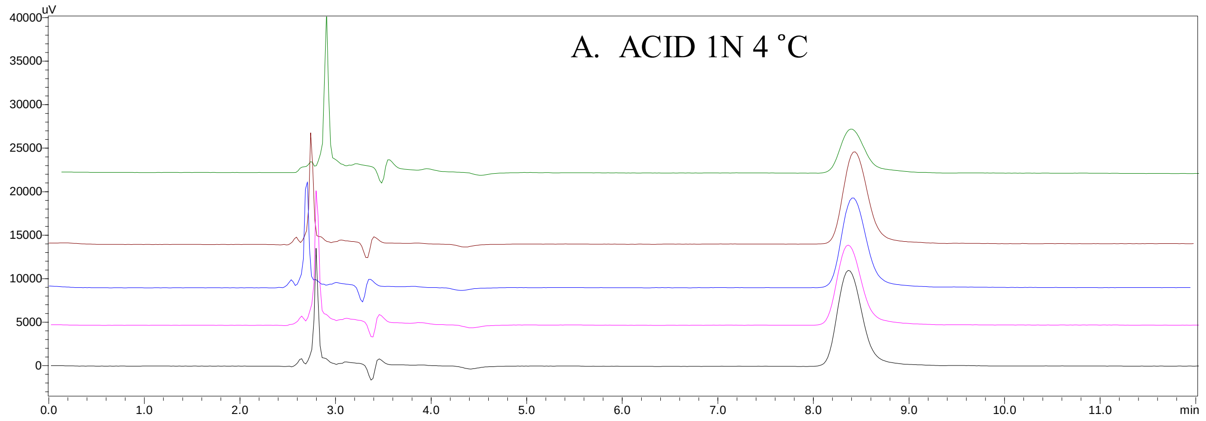
A. ACID 1N 4 ˚C
uV
40000
35000
30000
25000
20000
15000
10000
5000
0
0.0
1.0
2.0
3.0
4.0
5.0
6.0
7.0
8.0
9.0
10.0
11.0
min

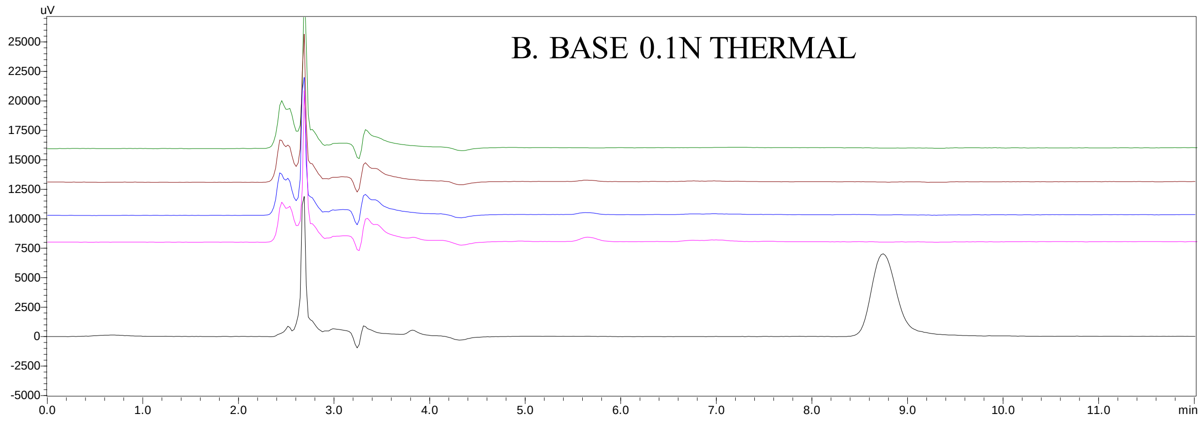
B. BASE 0.1N THERMAL
uV
25000
22500
20000
17500
15000
12500
10000
7500
5000
2500
0
-2500
-5000
0.0
1.0
2.0
3.0
4.0
5.0
6.0
7.0
8.0
9.0
10.0
11.0
min

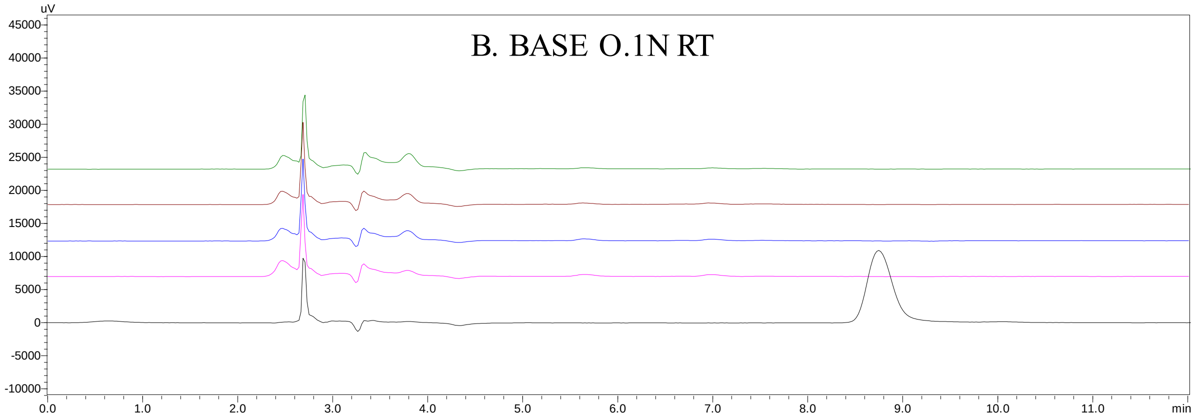
B. BASE O.1N RT
uV
45000
40000
35000
30000
25000
20000
15000
10000
5000
0
-5000
-10000
0.0
1.0
2.0
3.0
4.0
5.0
6.0
7.0
8.0
9.0
10.0
11.0
min

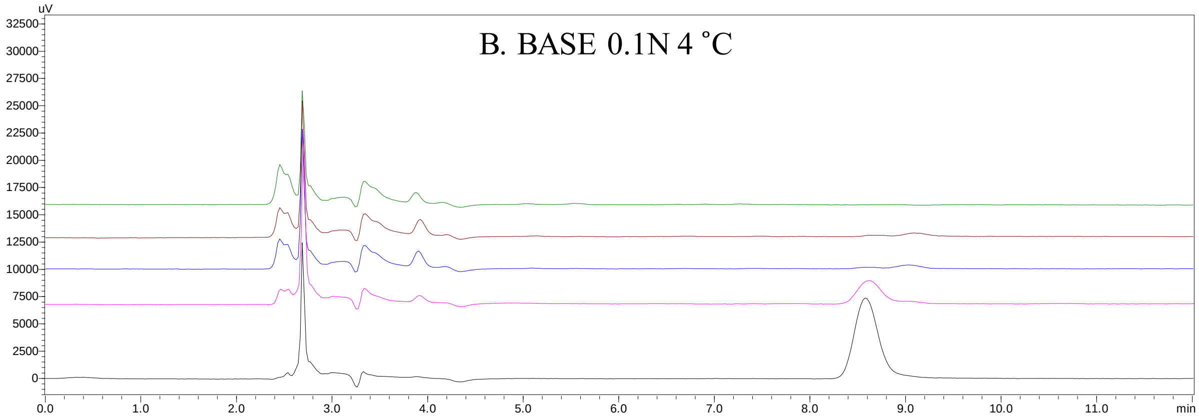
B. BASE 0.1N 4 ˚C
uV
32500
30000
27500
25000
22500
20000
17500
15000
12500
10000
7500
5000
2500
0
0.0
1.0
2.0
3.0
4.0
5.0
6.0
7.0
8.0
9.0
10.0
11.0
min

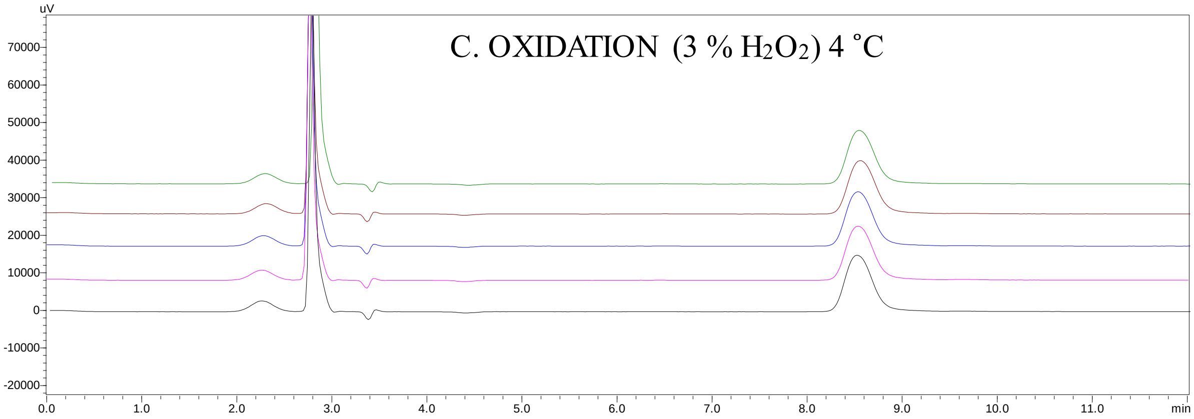
C. OXIDATION (3 % H_2O_2) 4 ˚C
uV
70000
60000
50000
40000
30000
20000
10000
0
-10000
-20000
0.0
1.0
2.0
3.0
4.0
5.0
6.0
7.0
8.0
9.0
10.0
11.0
min

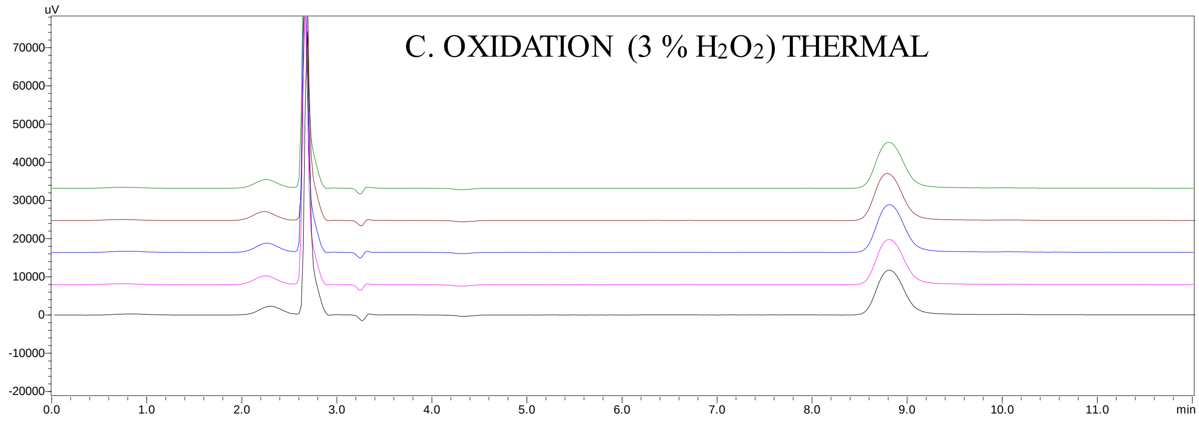
C. OXIDATION (3 % H2O2) THERMAL
uV
70000
60000
50000
40000
30000
20000
10000
0
-10000
-20000
0.0 1.0 2.0 3.0 4.0 5.0 6.0 7.0 8.0 9.0 10.0 11.0 min

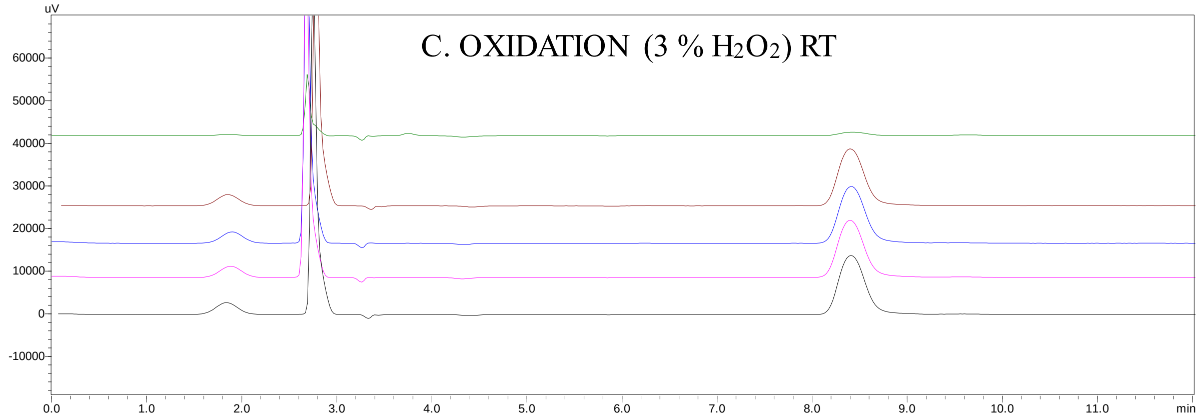
C. OXIDATION (3 % H2O2) RT
uV
60000
50000
40000
30000
20000
10000
0
-10000
0.0 1.0 2.0 3.0 4.0 5.0 6.0 7.0 8.0 9.0 10.0 11.0 min

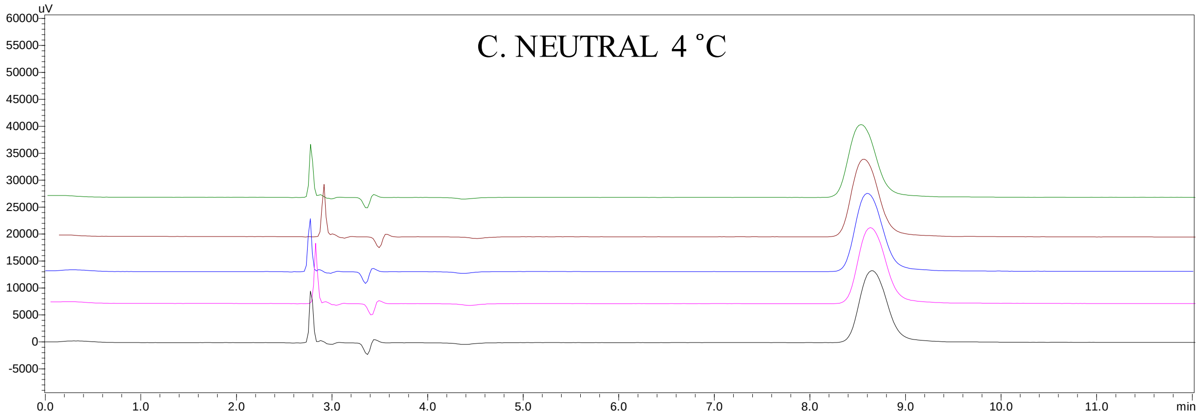
C. NEUTRAL 4 °C
uV
60000
55000
50000
45000
40000
35000
30000
25000
20000
15000
10000
5000
0
-5000
0.0 1.0 2.0 3.0 4.0 5.0 6.0 7.0 8.0 9.0 10.0 11.0 min

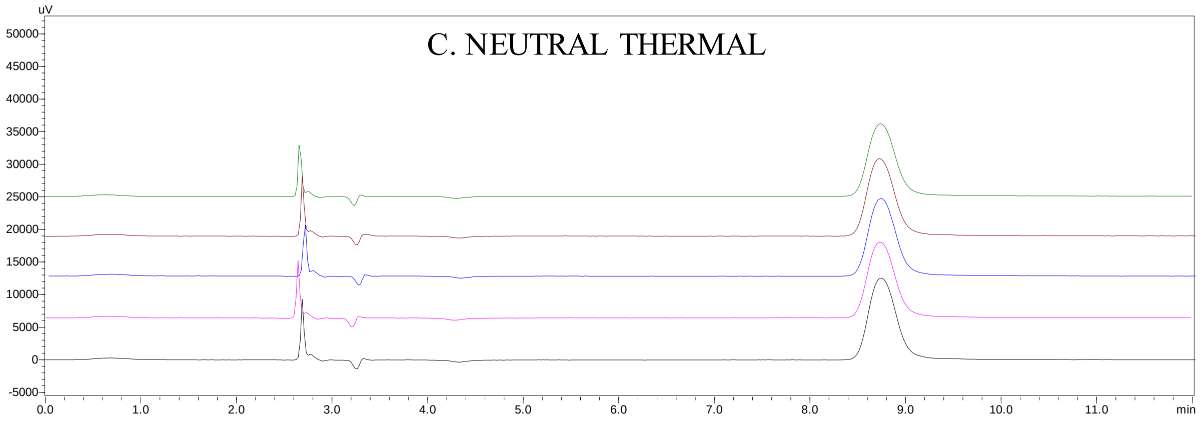
C. NEUTRAL THERMAL
uV
50000
45000
40000
35000
30000
25000
20000
15000
10000
5000
0
-5000
0.0
1.0
2.0
3.0
4.0
5.0
6.0
7.0
8.0
9.0
10.0
11.0
min

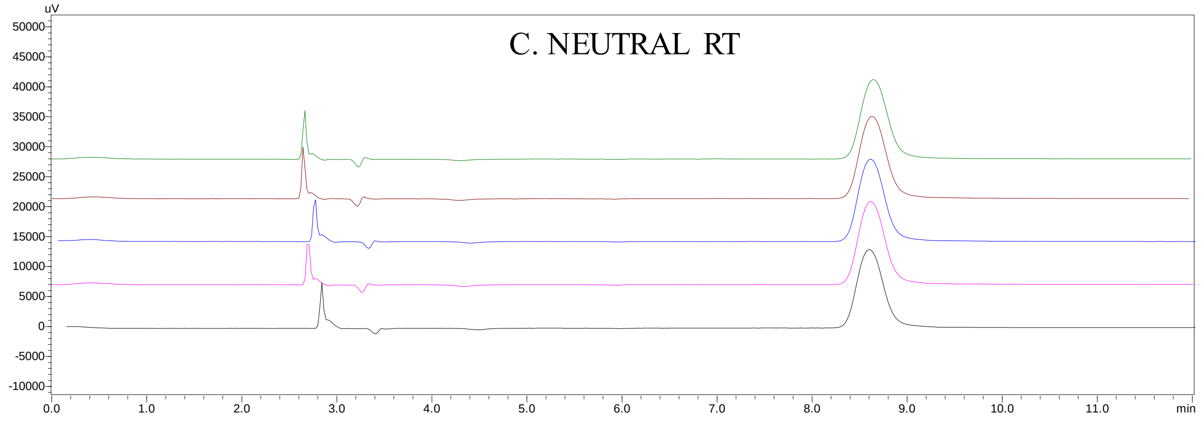
C. NEUTRAL RT
uV
50000
45000
40000
35000
30000
25000
20000
15000
10000
5000
0
-5000
-10000
0.0
1.0
2.0
3.0
4.0
5.0
6.0
7.0
8.0
9.0
10.0
11.0
min

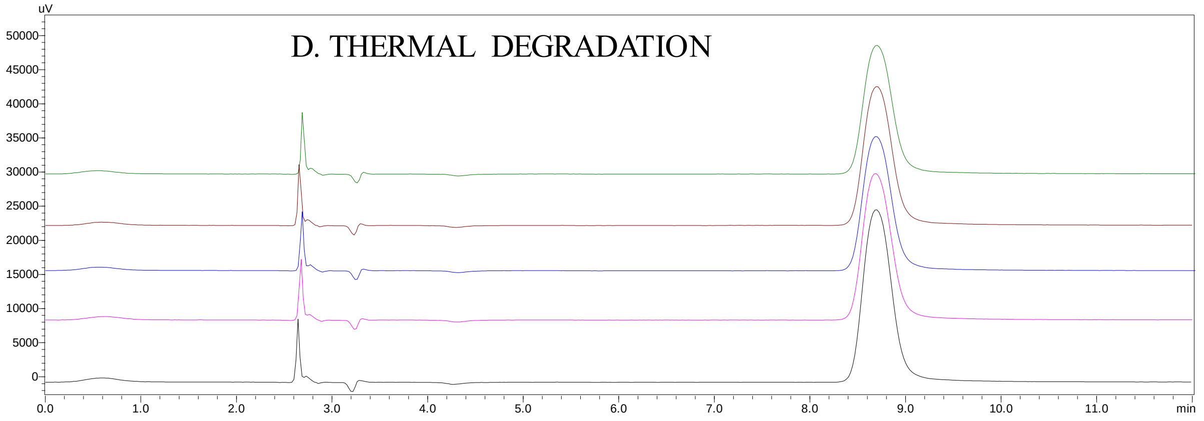
D. THERMAL DEGRADATION
uV
50000
45000
40000
35000
30000
25000
20000
15000
10000
5000
0
0.0
1.0
2.0
3.0
4.0
5.0
6.0
7.0
8.0
9.0
10.0
11.0
min

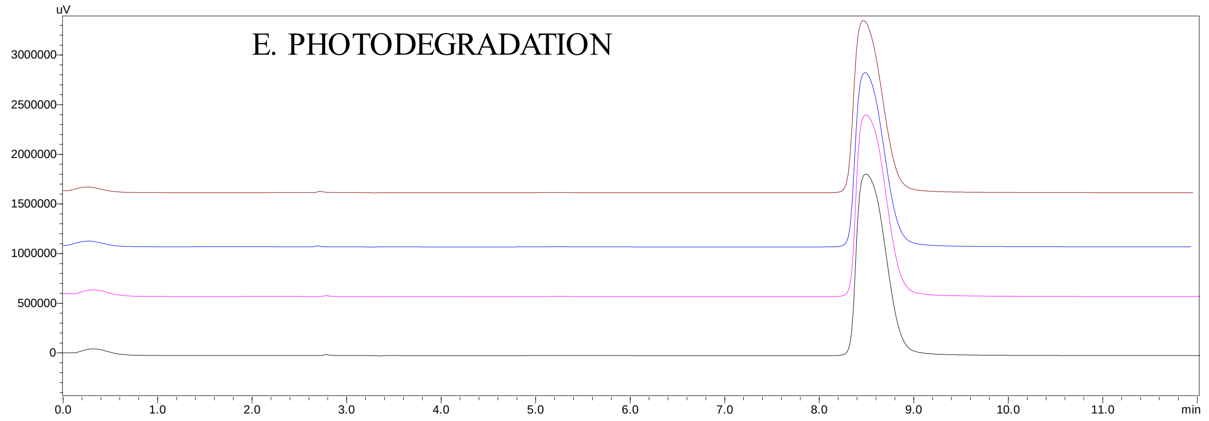

Figure s2: Overlay of HPLC chromatograms of Mangiferin when exposed to (A) 0.1 N and 1.0 N of HCl, (B) 0.1 N and 1.0 N of NaOH, (C) 3 % H_2O_2 oxidation and Neutral stress condition, (D) thermal degradation conditions and (E) photodegradation under different temperatures like 4 °C, RT and 60 °C.

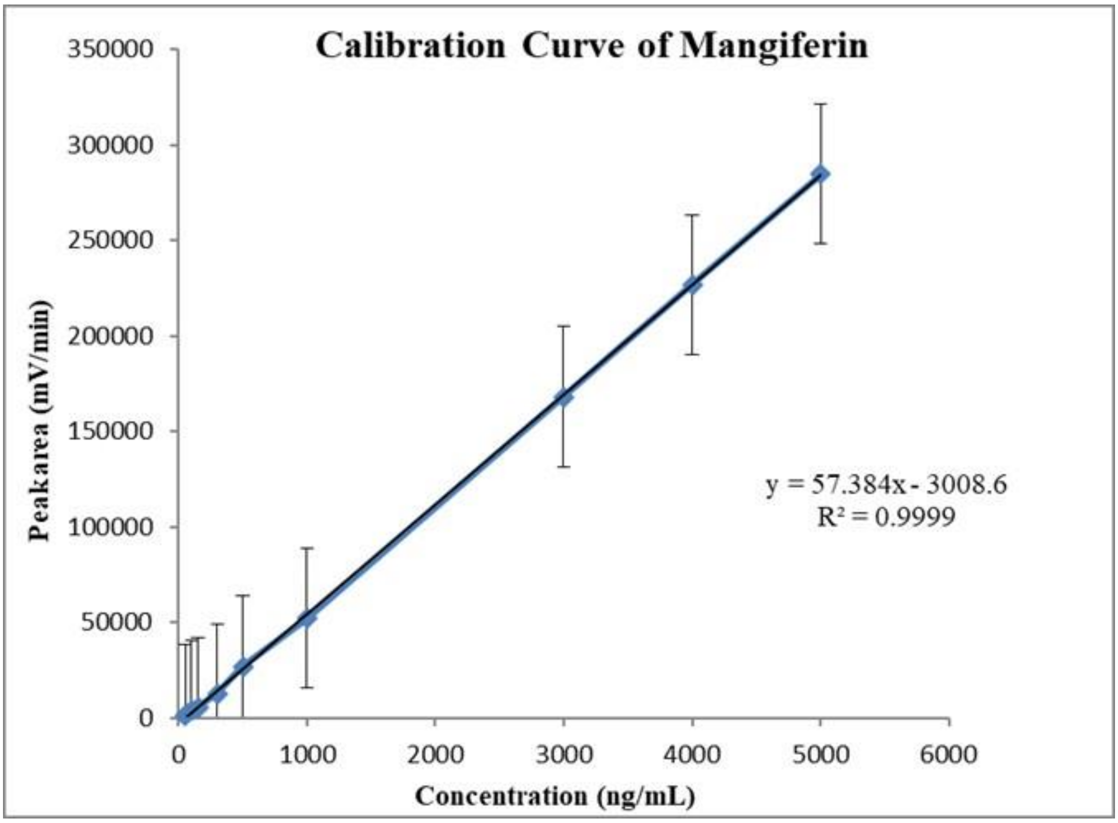

Figure s3: Calibration curve of Mangiferin.

Table s6: Precision data of Mangiferin for intra-day and inter-day

Intra-day				
**Concentration (ng/mL)**	**Response Factor = Peak area/ Conc**	**Average**	**SD**	**% RSD**
150	53.62	54.08	0.54	1.05
3000	54.68			
4000	53.93			
**Inter-day**				
**Concentration (ng/mL)**	**Response Factor = Peak area/ Conc**	**Average**	**SD**	**% RSD**
150	53.94	54.31	0.47	0.87
3000	54.84			
4000	54.15			
